# High‐Throughput Proteoform Imaging for Revealing Spatial‐Resolved Changes in Brain Tissues Associated with Alzheimer's Disease

**DOI:** 10.1002/advs.202416722

**Published:** 2025-03-12

**Authors:** Yue Sun, Dan Liu, Yu Liang, Xue Yang, Xinxin Liu, Baofeng Zhao, Zhen Liang, Yukui Zhang, Lihua Zhang

**Affiliations:** ^1^ State Key Laboratory of Medical Proteomics National Chromatographic R. & A. Center CAS Key Laboratory of Separation Science for Analytical Chemistry Dalian Institute of Chemical Physics Chinese Academy of Sciences 457 Zhongshan Road Dalian 116023 China; ^2^ University of Chinese Academy of Sciences Beijing 100049 China

**Keywords:** mass spectrometry imaging, proteoforms, spatial proteomics, throughput, top‐down proteomics

## Abstract

Spatially resolved characterization of proteoforms has substantial potential to significantly advance the understanding of physiological and disease mechanisms. However, challenges remain regarding throughput and coverage. A robust method is developed for high‐throughput proteoform imaging (HTPi) by combining matrix‐assisted laser desorption ionization mass spectrometry imaging (MALDI MSI) and region‐specific top‐down proteomic analysis. MALDI MSI enables the imaging of proteoforms on tissue sections at a rate of 7 h cm^−2^ (100‐µm spatial resolution), and the identification sensitivity of the proteoforms is improved by narrow‐bore monolithic columns with low adsorption, yielding 366 annotated proteoform images from the mouse brain. The obtained proteoform images reveals differential expression of individual proteoforms across the brain regions, and distinct spatial distribution patterns of various proteoforms generated from a single gene. Given its ability to visualize proteoform, HTPi is further applied to explore spatial pathological changes associated with Alzheimer's disease (AD) in 5 × FAD mice. 158 annotated proteoform images are obtained in hippocampal regions at 50‐µm spatial resolution, illuminating 14 differential proteoforms in the subiculum region and highlighting their significant associations with amyloid‐β pathology in AD. The results highlight the power of HTPi in unraveling the intricate molecular landscape of brain tissues and its potential in elucidating disease mechanisms.

## Graphical abstract

High‐throughput proteoform imaging (HTPi) is established by combining matrix‐assisted laser desorption ionization mass spectrometry imaging and high‐sensitivity region‐specific top‐down proteomic analysis. HTPi enables the generation of annotated proteoform images with exceptional precision, high throughput, and deep coverage, showing distinct spatial distribution patterns of various proteoforms and further revealing spatially resolved changes in brain tissues associated with Alzheimer's disease.

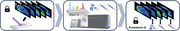

## 1 Introduction

Biological tissues, such as the brain, exhibit intricate and diverse functionalities through their highly partitioned structural regions.^[^ ^1^ ^]^ Their complex phenotypes manifest as molecular variety and spatiotemporal heterogeneity of proteome across functional tissue units. Nevertheless, conventional proteomics analysis of bulk tissues leads to the loss of spatial information. Notably, given the proteome changes involved in disease states,^[^ ^2^ ^]^ this absence of spatial information also limits our comprehension of disease mechanisms.

Several methods based on bottom‐up strategies and laser capture microdissection (LCM) have been developed to determine the spatial heterogeneity of the proteome.^[^ ^3^ ^]^ However, these spatial proteomic analysis methods have focused primarily on the protein‐coding gene level,^[^ ^4^ ^]^ overlooking proteoform‐level information. Proteoforms are different forms of proteins that arise from collective biological processes, including amino acid variations, alternative RNA splicing, posttranslational modifications (PTMs), and truncations.^[^ ^5^ ^]^ The proteoforms produced by a single gene have been shown to exhibit different tissue‐specific localizations and functions. For example, three methylated proteoforms of cysteine‐rich protein 1 (CRIP1) showed significantly different spatial distributions in tumor and stromal regions in ovarian cancer tissue.^[^ ^6^ ^]^ The higher relative level of unmethylated CRIP1 in vascularized locations in the stroma might be related to its functional role in angiogenesis. Therefore, investigating proteoforms, particularly when incorporating spatial information, holds immense potential to uncover more precise biological mechanisms. Thus, it is imperative to develop tools that enable spatially resolved proteoform analysis.

Considerable research efforts have been made in the spatial‐resolved analysis of proteoforms via LCM‐based methods or in situ mass spectrometry imaging (MSI)‐based approaches. LCM coupled with top‐down proteomic analysis^[^ ^7^ ^]^ is suitable for identifying proteoforms for specific regions of interest (ROIs), however, it is difficult to achieve pixel‐by‐pixel imaging of proteoforms on tissue sections with high spatial resolution because of the time‐consuming analysis of thousands of LCM slices.

In contrast, current MSI‐based approaches, such as nanospray desorption electrospray ionization (nano‐DESI) and matrix‐assisted laser desorption ionization (MALDI), can generate proteoform images from tissue sections in situ. Kelleher et al. used nano‐DESI to perform proteoform imaging on tissue sections at 150 µm spatial resolution with a scan rate of 4.6 h cm^−2^. On‐tissue top‐down proteomics experiments were conducted on the MSI‐adjacent section to identify proteoforms, allowing the identification of 25 targeted proteoforms from nano‐DESI mass spectrometry (MS) images.^[^ ^8a^ ^]^ When a 20 µm spatial resolution was used, the scan rate was decreased to 55.5 h cm^−2^. An intact mass tag was used to search against custom proteoform databases to improve proteoform coverage to improve proteoform coverage, enabling the annotation of ≈300 proteoforms.^[^ ^6^ ^]^ MALDI MSI represents an appealing alternative for proteoform imaging.^[^ ^9^ ^]^ The robust ionization performance of lasers in a vacuum ensures the stable detection of biological molecules from tissues. Given the high throughput of the time‐of‐flight (TOF) analyzer, a single MALDI MSI experiment has enabled the production of hundreds of protein images at 30 µm spatial resolution with a scan rate of 1.2 h cm^−2^.^[^ ^10^ ^]^ Despite all these advantages, the gas‐phase fragmentation efficiency of MALDI‐generated proteins is low, making it difficult to identify proteoforms from tissues directly. Alternatively, researchers have employed techniques such as liquid microjunction or parafilm‐assisted microdissection to acquire proteomic samples from tissue sections for independent proteoform identification to achieve MSI annotation.^[^ ^9^, ^11^ ^]^ However, up to now, MALDI MSI still suffers from low proteoform coverage with clear annotation, thereby limiting further applications in analyzing complex biological samples.

In this work, to overcome the challenges of MALDI MSI, a region‐specific top‐down proteomics strategy with deep coverage was integrated as a part of MSI workflows to provide accurate proteoform confirmation. Narrow‐bore monolithic columns with low adsorption were developed for proteoform separation to increase the sensitivity of top‐down proteomic analysis for limited regions, which were microdissected from the tissue section adjacent to the MSI used. The identified proteoforms from selected ROIs were used to match peaks generated from the same ROIs in the MSI data, reducing the matching error probability caused by the neglect of tissue heterogeneity. In conjunction with MALDI MSI, the high‐throughput proteoform imaging (HTPi) workflow was successfully established, exhibiting exceptional precision, high throughput, and deep coverage. This robust workflow enabled the investigation of proteoform distribution patterns across different brain regions, revealing proteoform perturbation in the hippocampus (HP), cortex (CTX), thalamus (TH), and striatum (ST) of brain tissues in 5×FAD mice. HTPi was further applied to explore the distribution of proteoforms in subregions of the HP, highlighting significant links between proteoforms and amyloid‐β (Aβ) pathology in Alzheimer's disease (AD).

## 2 Results and Discussion

### 2.1 High‐Sensitivity Top‐Down Method for Proteoform Identification

To overcome the deficiencies in proteoform identification by MALDI MSI, a region‐specific top‐down method was developed using C8‐functional amine‐bridged hybrid capillary monolithic columns (TEOOS‐BTMSPA). In our previous work,^[^ ^12^ ^]^ monoliths with unique macroporous structures and bridged secondary amino groups were demonstrated to exhibit advantages in reduced mass transfer resistance and low nonspecific adsorption to proteins, which would be beneficial for the analysis of limited protein samples. In this work, the inner diameter of the monolith was reduced to 50 µm with a low flow rate of 120 nL min^−1^ for nanoflow reversed‐phase liquid chromatography‐tandem mass spectrometry (RPLC‐MS/MS) experiments, under which conditions, carbonic anhydrase could be detected at a concentration as low as 3.3 fmol (Figure S1, Supporting Information). The MS signal intensity of proteoforms from the mouse brain tissue sections could be increased by fivefold compared with that of conventional nanoflow separation using 100 µm‐i.d. columns with a flow rate of 500 nL min^−1^ (Table S3, Supporting Information). In total, 406 and 236 proteoforms were identified from tissue sections with areas of 0.5 and 0.1 mm^2^ (Table S4, Supporting Information), respectively, achieving over fivefold proteoform coverage compared with the previously reported results of conventional RPLC‐MS/MS.^[^ ^9^, ^11^, ^13^ ^]^ This method was further applied to study LCM‐derived mouse brain sections from the CTX, HP, TH, and ST regions, identifying 630, 788, 478, and 486 proteoforms (Figure S2, Supporting Information), respectively, which subsequently served as a spatially resolved proteoform database for assigning signals in the MALDI MSI data.

### 2.2 Mouse Brain Proteoforms Visualized Using HTPi

**Figure 1:**
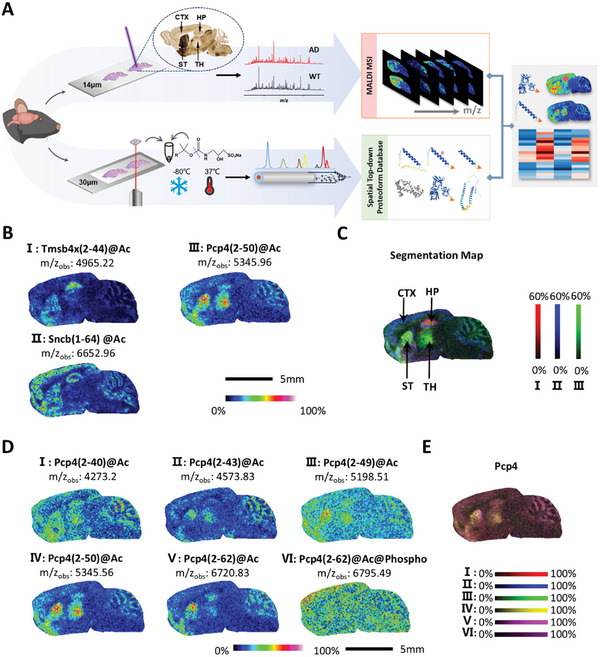
HTPi workflow and proteoform imaging of tissue sections from 6‐month‐old wild type mouse brain tissues. A) HTPi workflow combining MALDI MSI and high‐sensitivity region‐specific top‐down proteomic analysis. B) Region‐specific expression proteoform images. C) Segmentation map of the mouse brain using the proteoforms images in (B). D) MALDI‐MSI images of six proteoforms of Pcp4. E) Integrated MSI image of Pcp4 proteoforms.

HTPi achieves visualization of proteoforms in mouse brain tissues by combining MALDI MSI and high‐sensitivity region‐specific top‐down proteomics (**Figure** **1**A). Specifically, ultrafleX MALDI was used to image 14‐µm‐thick sections from mouse brains at the proteoform level with a raster width of 100 µm. The high throughput of data collection permitted the generation of an MSI dataset containing 5567 pixels within 4 h of acquisition, corresponding to a rate of 7 h cm^−2^. For accurate MALDI MSI annotations, proteoform identification was performed using region‐specific top‐down tandem mass spectrometry (MS/MS) data acquired from a 30‐µm‐thick section adjacent to the MSI used. MSI peaks above 1% relative abundance were selected from the total ion current (TIC) of CTX, HP, TH, and ST and assigned from their corresponding region‐specific top‐down datasets within ± 0.05% mass tolerance. As a result, 366 proteoforms with MS images were annotated (Tables S3 and S4, Supporting Information), including 293 truncated proteoforms and 213 proteoforms containing PTMs, such as acetylation, methylation, phosphorylation, and oxidation.

The proteoform images showed typical spatial distribution heterogeneity, which was highly correlated with the morphological segmentation of the brain regions. As illustrated in Figure 1B, Tmsb4x(2–44)@Ac was expressed mainly in the HP and ST regions, and Pcp4(2–50)@Ac was expressed mainly in the ST and TH regions. Cell type‐related information was also observed, such as that Sncb(1–64)@Ac was highly abundant only in the granule cells of the HP region. These three region‐specific expressed proteoforms were used to present spatial segmentation on the tissue section of the mouse brain (Figure 1C), which was also used to select the ROIs. Furthermore, multiple proteoforms generated from a single gene exhibited specific spatial distributions. For example, six proteoforms of the calmodulin regulator protein PCP4 (Pcp4), a key protein in the development and functioning of the nervous system, were imaged with different distribution patterns in ST, TH, and CTX (Figure 1D). Pcp4(2–62)@Ac was present mainly in the ST region, the truncated Pcp4 proteoforms were mainly distributed in ST and TH regions, and Pcp4(2–62)@Ac@Phosphowas was highly abundant in the CTX region. The integrated MSI image of these Pcp4 proteoforms (Figure 1E) was consistent with the results obtained via in situ hybridization imaging^[^ ^14^ ^]^ and immunofluorescence (Figure S3, Supporting Information), which allowed for the examination of Pcp4 distribution at the gene and protein group levels, respectively. Our findings have expanded the application of spatial visualization to the proteoform level.

### 2.3 HTPi Spatially Resolved Proteoform Characterization in 5×FAD Mice

**Figure 2:**
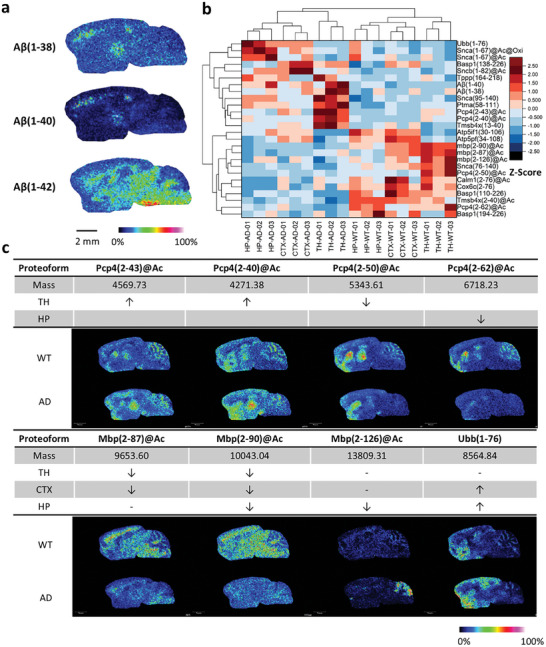
Spatial‐resolved characterization of proteoforms in brain tissue slices from WT and AD mice. A) MS images of Aβ proteoforms in the AD mouse brain. B) Heatmap of regulated proteoforms (*y*‐axis) quantified between WT and AD samples (*x*‐axis). C) Proteoform images of Pcp4, Mbp, and Ubb show significant differences between WT and AD mouse brains. Ion images of the proteoforms normalized to the TIC.

HTPi was further applied to investigate proteoform perturbation associated with AD in 5×FAD mice. As shown in Figure S4A,B (Supporting Information), proteoforms were quantified according to MSI peak intensities in different brain regions from wild type (WT) and AD mice with three biological replicates. The apparent differences were mainly reflected in HP, CTX, and TH (Figure S4B, Supporting Information). Aβ proteoforms were detected in these three regions in the AD state (**Figure** **2**A), which is consistent with the thioflavin S (ThS) staining localization of aggregates in AD brain tissue sections (Figure S4C, Supporting Information). Therefore, subsequent analyses focused on the regions of HP, CTX, and TH regions.

Proteoforms with highly differential abundance in these three brain regions were filtered out according to the MSI dataset and annotated with region‐specific top‐down data (Figure S5, Supporting Information). As a result, 26 identified proteoforms presented spatially differential abundances between WT and AD; furthermore, according to the clustering results, these proteoforms highly correlated with changes in the CTX and HP regions (Figure 2B). Images with annotations are displayed in Figure 2C and Figure S5D (Supporting Information). The regulation of proteoforms, such as Ubb(1–76), Snca(1–67)@Ac, and Snca(1–67)@Ac@Oxi, presented a similar trend in the CTX and HP regions. Furthermore, various proteoforms derived from a single gene underwent different alterations in specific regions. Four of the six Pcp4 proteoforms found in the WT mouse brain exhibited differential abundances in the AD state, as reflected by the downregulation of Pcp4(2–62)@Ac in the HP region and Pcp4(2–50)@Ac in the TH region accompanied by the upregulation of Pcp4(2–43)@Ac and Pcp4(2–40)@Ac in the TH region (Figure 2C). In a previous study, Pcp4 was demonstrated to increase Aβ deposition and aggravate AD‐related phenotypes but was detected only at the protein level using western blotting.^[^ ^15^ ^]^ The observed trend in the abundance of Pcp4 proteoforms and their colocalization with Aβ in our findings suggest that Pcp4 may undergo truncation during its involvement in the process of Aβ aggregation. We also observed reduced Mbp(2–87)@Ac in the TH and CTX regions, Mbp(2–90)@Ac in the TH, CTX and HP regions, and Mbp(1–126)@Ac in the HP regions. These proteoforms of myelin basic protein (Mbp) play a role in the formation and stabilization of the myelin membrane. Myelin dysfunction was reported to cause the accumulation of the Aβ‐producing machinery within axonal swellings and increase the cleavage of the cortical amyloid precursor protein in 5×FAD mice with demyelination, which indicated the disruption of myelin integrity was a potential upstream risk factor for Aβ deposition.^[^ ^16^ ^]^ Therefore, the overall reduction of Mbp proteoforms may be related to myelin dysfunction, which affects Aβ deposition. Additionally, Ubb(1–76) was upregulated in the CTX and HP regions of AD tissues, which might be associated with the ubiquitin–proteasome system (UPS). UPS plays a decisive role in Aβ clearance, and its dysfunction leads to the accumulation of Aβ and the formation of plaques in the pathological process of AD.^[^ ^17^ ^]^ These findings enhance our understanding of Aβ pathology in AD and provide insights into potential novel therapeutic targets for AD at the proteoform level.

**Figure 3:**
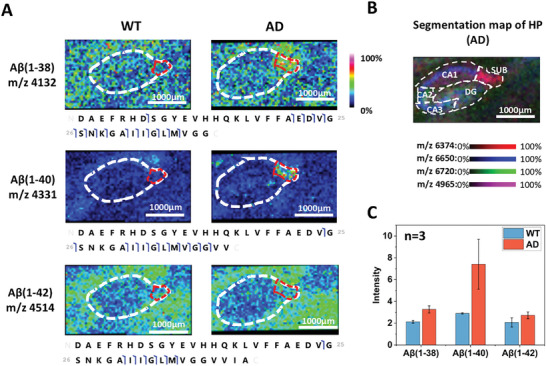
Detection of Aβ proteoforms in the SUB. A) MS images and top‐down identified sequences of Aβ(1–38), Aβ(1–40), and Aβ(1–42). B) Segmentation map of the HP region. C) Comparison of Aβ proteoforms’ intensity in the SUB region between WT and AD mice. The intensity was normalized to the TIC of the SUB region. Data are shown as mean ± SD (n = 3).

The HP is a complex and heterogeneous structure comprising multiple subregions with distinct structures and functions: the dentate gyrus (DG), cornu ammonis fields CA1, CA2, CA3, and the subiculum (SUB).^[^ ^18^ ^]^ As each subregion performs different functions, the pathological changes that occur during AD progression also vary. To prevent signal dilution caused by studying the HP as a whole, we performed MS imaging targeting the HP region at a higher resolution with proteoform annotation using subregion‐specific top‐down datasets. As a result, 158 proteoforms were imaged at the resolution of 50 µm in the HP to investigate AD pathology at the subregion level, as well as 98 proteoforms at the resolution of 20 µm. Distinct Aβ species have been reported to play critical roles in AD‐related biological mechanisms. In this study, we observed that three Aβ proteoforms exhibited high abundance exclusively in the SUB region of the HP in 5×FAD mice (**Figures** **3** and S6, Supporting Information), which aligns with the localization of aggregates (Figure S4C, Supporting Information). These findings suggest that the SUB serves as a key region in the progression of AD, as Aβ aggregation is a significant pathological hallmark of AD.

**Figure 4:**
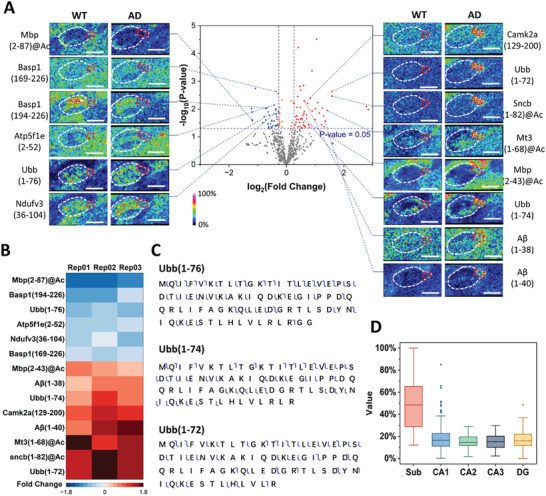
Analysis of differential proteoforms in the SUB. A) Volcano plot (middle) generated from label‐free quantitation using the MSI dataset of the SUB from WT and AD samples. HTPi images of six downregulated proteoforms (left) and eight upregulated proteoforms (right) are correlated with their quantitation outcome indicated by dashed lines in the volcano plot. Proteoforms labeled in the volcano plot are annotated by the SUB region‐specific top‐down MS/MS data. N = 3, *P* < 0.05, unpaired two‐tailed t test. B) The reproducibility of quantitation for the 14 differentially detected proteoforms is highlighted in (A). C) Top‐down identification of Ubb proteoforms. D) Box plots of intensity values for Ubb (1–72) at all spots within the segmented HP subregions in the AD section. The *y*‐axis represented relative intensity.

As illustrated in **Figure** **4**A,B, six downregulated and eight upregulated proteoforms were observed in the SUB. Ndufv3(36–104) and Atp5f1e(2–52) are components of the mitochondrial membrane. Their downregulation may be associated with mitochondrial dysfunction in AD, as Aβ can interact with mitochondrial components and exert cellular toxicity.^[^ ^19^ ^]^ In addition, the differentially abundant polyubiquitin‐B (Ubb) proteoforms are highlighted in Figure 4C. As mentioned previously, Ubb(1–76) upregulation was observed in the HP region. Herein, when observing the subregions within the HP region, Ubb(1–76) was downregulated in the SUB. Simultaneously, the levels of the truncated proteoforms Ubb(1–74) and Ubb(1–72) were found to be increased in the SUB. These two inactivated Ubb fragments are generated through the cleavage of Ubb by insulin‐degrading enzyme (IDE), with the rapid removal of the two C‐terminal glycines followed by slow cleavage between residues 72 and 73.^[^ ^20^ ^]^ Given that IDE has been revealed as an enzyme that can participate in the extracellular degradation of Aβ in vivo,^[^ ^21^ ^]^ the increased affinity of Ubb(1–72) for IDE may hinder IDE‐mediated Aβ clearance, thereby facilitating the formation of Aβ aggregates. Additionally, Ubb(1–72) was observed only in the SUB (Figure 4D), indicating that the pathological process described above occurred specifically in the SUB, consistent with the spatial distribution of aggregates in the ThS staining results (Figure S4C, Supporting Information). Our investigation of spatially resolved proteoforms indicates promising potential for providing a novel interpretation of Aβ homeostasis occurring in AD.

## 3 Conclusion

We established a robust workflow, HTPi, to uncover spatial heterogeneity at the proteoform level with exceptional precision, high throughput and deep coverage. Given the spatial heterogeneity of brain tissue, the obtained proteoform images revealed that the spatial distribution of proteoforms was associated with their function. Furthermore, spatially resolved pathological changes in brain tissue sections from an AD mouse model were explored, highlighting significant associations between differential proteoforms and Aβ pathology in AD. Be noted that, due to signal inhibition by high‐molecular‐weight proteoforms, its current molecular weight detection range is limited to less than 20 kDa. Therefore, the further improvement will be high‐molecular‐weight proteoform imaging. Future applications of HTPi include but are not limited to imaging proteoforms at a brain‐wide scale in 3D and the integration of temporal and spatial information to develop multi‐scale techniques for biological characterization of tissues. Besides, HTPi holds significant clinical potentials, such as biomarker discovery by analyzing specially‐resolved expression differences of proteoforms between diseased and healthy tissues, and disease diagnosis and classification by imaging tumor‐specific proteoform markers.

## 4 Experimental Section

### Chemicals and Reagents

Ultrapure water (18.2 MΩ cm) was produced via a Milli‐Q system from Millipore (Milford, MA, USA). The cleavable detergent ProteaseMAX was obtained from Promega (Madison, WI, USA). n‐Dodecyl β‐D‐maltoside (DDM) was purchased from Aladdin Chemistry (Shanghai, China). Pluronic® F‐127 (F127), bis[3‐(triethoxysilyl)propyl]amine (BTMSPA, technical grade, ≥90%), triethoxy(octyl)silane (TEOOS, 97.5%), protease inhibitor cocktail (for use with mammalian cell and tissue extracts, DMSO solution), ribonuclease A, cytochrome c, myoglobin, and carbonic anhydrase were obtained from Sigma‐Aldrich (St.Louis, MO, USA). Hexadecyltrimethylammonium chloride (C_16_TACl) was purchased from J&K Scientific (Beijing, China).

### Tissue Preparation

The 6‐month‐old male C57BL/6 and 5 × FAD mice (Mus musculus) used in this study were sourced from Shulaibao Biotechnology (Wuhan, China). All procedures were approved by the Animal Care and Use Committee (ACUC) of Wuhan Youdu Biotechnology Co. Ltd., China (Approval Number: 20 230 510). The mice were anesthetized via the injection of sodium pentobarbital and then exsanguinated until death. The brain tissues were dissected, frozen on dry ice, and stored at −80 °C until use.

The frozen brain samples were sectioned using a cryostat microtome (Leica CM1860, Leica Microsystems, Wetzlar, Germany). Sagittal brain sections (lateral 1.2 mm) were prepared with reference to the Allen Brain Reference Atlases from http://atlas.brain‐map.org/. For the MALDI MSI experiments, the brain sections were sliced to a thickness of 14 µm at −20 °C. The tissue sections were thaw‐mounted onto indium tin oxide (ITO)‐coated glass slides (Bruker Daltonics, Bremen, Germany). The adjacent tissue sections were cut at a thickness of 30 µm and mounted on polyethylene naphthalate membrane slides (Leica, Heidelberg, Germany). After tissue section preparation, the slides were immediately dehydrated under vacuum at room temperature for 20 min and stored at −80 °C until use.

### MALDI MSI Analysis

Before matrix coating, serial parallel tissue sections were rinsed six times using the following protocol: 70% ethanol, 100% ethanol, Carnoy's mixture (60% ethanol, 30% dichloromethane, and 10% acetic acid), 100% ethanol, 0.2% trifluoroacetic acid (TFA), and 100% ethanol (% represents volume ratios without no special instructions). All rinsing steps were performed for 30 s, except for the step with Carnoy's solution, which was applied for 2 min. Following washing, the ITO slides were dried in a desiccator for 20 min before being coated with the matrix. Three milliliters of α‐cyano‐4‐hydroxycinnamic acid (7 mg mL^−1^ in 60% ACN, 0.2% TFA) were coated automatically on the brain tissue section using an automatic sprayer (ImagePrep 2.0, Bruker Daltonics, Bremen, Germany). The matrix was sprayed over 30 layers (60 s drying time between each layer).

MALDI MSI experiments were performed using a MALDI‐TOF/TOF (ultrafleX, Bruker Daltonics, Bremen, Germany) mass spectrometer equipped with a Smartbeam II 2 kHz laser and operated in positive‐ion mode. The laser power was optimized at the start of each run and then held constant during the MALDI MSI experiment. For the whole brain section and HP region, the laser focus was set to large for 100 and 50 µm raster width analysis, respectively. At each sampling position, 500 shots were used to acquire data for the m/z 2500–20000 range. The methods used were externally calibrated using protein calibration standard I (Bruker Daltonics, Bremen, Germany).

### Preparation of TEOOS‐BTMSPA monoliths

The capillary (50 µm i.d./360µm o.d.) was sequentially flushed with 1 M hydrochloric acid, water, 4% hydrofluoric acid, water, 1 M sodium hydroxide, water, and methanol. The capillary was subsequently dried at 120 °C under a nitrogen atmosphere overnight. A homogeneous reaction mixture was prepared by mixing 24 mg of C_16_TACl, 18 mg of F127, 200 µL of methanol, 25 µL of TEOOS, 25 µL of BTMSPA and, 20 µL of water thoroughly, and then immediately pumping them into the pretreated capillary. With both ends of the capillary sealed by rubber, the sol‒gel reaction was performed at 40 °C for 12 h. Subsequently, the column was rinsed with methanol to remove unreacted reagents.

### LCM and Sample Preparation

Brain tissue sections were fixed and dehydrated by immersing them successively in 70% ethanol, 100% ethanol, Carnoy's fluid (ethanol: dichloromethane, 2:1), and 100% ethanol. After tissue section preparation, the slides were immediately dehydrated under vacuum at room temperature for 20 min and stored at −80 °C until use. A PALM MicroBeam system (Carl Zeiss MicroImaging, Munich, Germany) was employed to cut areas of the brain tissue. First, sections (0.1 or 0.5 mm^2^ × 30 µm each) were collected for evaluation of the top‐down workflow. For 6‐month‐old WT and AD mice, three sections (0.1 mm^2^ × 30 µm each) from each specific region were collected into a single tube, targeting CTX, TH, and ST regions. Specially, sections of DG, CA1, CA2, CA3, and SUB sections from the HP region were collected separately. The LCM sections were stored at −80 °C before further sample preparation for top‐down analysis.

Protein extraction from LCM‐derived sections was performed using a repeated freeze/thaw protocol in 4.5 µL of the lysis buffer containing 0.1% (w/v) ProteaseMAX, 0.01% (w/v) DDM, 1% (v/v) protease inhibitor cocktail, and 10 mM NH_4_HCO_3_ (pH 8). In detail, the tubes containing samples were placed into a −80 °C freezer for 5 min and thawed at 37 °C for 2 min for a total of five rounds of the freeze–thaw cycles. After the last thaw, the samples were acidified by adding 0.5 µL of 20% (v/v) formic acid and incubated at 37 °C for 15 min.

### NanoRPLC‐MS/MS Analysis

The nanoflow RPLC‐MS/MS was performed on an Orbitrap Fusion Lumos coupled with an Easynano 1200 LC system (Thermo Scientific). The samples were directly loaded onto a 30 cm × 50 µm i.d. TEOOS‐BTMSPA monolith, followed by binary gradient separation. Mobile phase A comprised 96% H_2_O, 2% acetonitrile, and 2% formic acid, and mobile phase B included 18% H_2_O, 80% acetonitrile, and 2% formic acid. The flow rate was 120 nL min^−1^. MS1 spectra were obtained with a resolution of 60000, two microscans, an automatic gain control (AGC) target of 5 × 10^5^, a maximum injection time of 50 ms, and a scan range of 600–2000. The three most abundant ions with charge states greater than +4 were selected using an isolation window of 5 m/z. The selected precursors were then fragmented through higher‐energy collision dissociation fragmentation with 20% collision energy. MS2 spectra were obtained with a resolution of 60000, two microscans, an AGC target of 5 × 10^5^, and a maximum injection time of 400 ms.

### Data Processing and Statistical Analysis

For top‐down analysis, the RAW files were converted into mzML files using the MSConvert tool. The TopFD tool was employed for spectral deconvolution. The resulting msalign files were then processed using TopPIC (v1.6.1) for database searching against UniProtKB databases of Mus musculus. The maximum number of mass shifts was set to 1. The precursor and fragment mass error tolerances were set to 15 ppm, and proteoforms with similar masses were merged by setting the proteoform‐spectrum match cluster error tolerance to 3 Da. The database search results were filtered with a 1% proteoform‐level false discovery rate (FDR) and assessed using the target‐decoy approach. The data acquired in “high‐high” mode were also analyzed using Proteome Discoverer (Thermo Scientific, version 2.5) equipped with the ProsightPD 4.0 node. The deconvolution of the MS1 and MS2 spectra was performed using the Xtract algorithm. For the database search, a three‐tiered search, comprising an absolute mass search with narrow precursor tolerance (2.2 Da), a biomarker search (10 ppm precursor tolerance), and an absolute mass search with wide precursor tolerance (100 Da) plus delta mode option, was used (with the three searches running simultaneously). The mass tolerance for fragment ions was 10 ppm. The FDR cutoff was set as 1% at the proteoform level. The generated proteoform dataset was combined manually. The MALDI MSI data files were imported into flexImaging (Bruker Daltonics, v.4.0). The processing of TIC normalization was complete for the generation of ion images. Several ion images with distinct brain region‐specific distributions were used to segment the ROIs manually. The intensity was normalized to the TIC of ROIs and exported to perform peak picking using a 1% relative abundance threshold and the peaks were annotated using spatially resolved proteoform databases of their corresponding regions within ±0.05% mass tolerance. The data was presented as the mean ± SD from three experiments. For quantitative analysis of proteoforms, the significant difference was determined by an unpaired two‐tailed t test. The proteoforms in the sample group with an intensity change of over 1.2 fold and p < 0.05 compared to the control group were marked as significantly different proteoforms.

### Ethical Statement

All procedures of animal experiments were conducted in accordance with the “Guiding Principles in the Care and Use of Animals” (China) and were approved by ACUC of Wuhan Youdu Biotechnology Co. LTD., China (Approval Number: 20 230 510).

## Conflict of Interest

The authors declare no conflict of interest.

## Author Contributions

Y.S. and D.L. contributed equally to this work. Y.S. did conceptualization, methodology, formal analysis, investigation, resources, data curation, wrote the original draft, review & editing, and visualization. D.L. did conceptualization, methodology, formal analysis, investigation, data curation, reviewing & editing, and visualization. Y.L. did conceptualization, methodology, funding acquisition, supervision, reviewing & editing. X.Y. performed a formal analysis and investigation. X.L. did an investigation. B.Z. and Z.L. did supervision, review and editing. Y.Z. performed funding acquisition and supervision. L.Z. did conceptualization, funding acquisition, supervision, reviewing & editing.

## Supporting information



Supporting Information

Supplemental Table S3

Supplemental Table S4

## Data Availability

The mass spectrometry proteomics data have been deposited to the ProteomeXchange Consortium (http://proteomecentral.proteomexchange.org) via the iProX partner repository^[^ ^22^ ^]^ with the dataset identifier PXD058552.

## References

[1] a) T. R. Cox , J. T. Erler , Dis. Model. Mech. 2011, 4, 165; 21324931 10.1242/dmm.004077 PMC3046088

[2] T. R. Cox , Nat. Rev. Cancer 2021, 21, 217. 33589810 10.1038/s41568-020-00329-7

[3] a) A. Mund , A. D. Brunner , M. Mann , Mol. Cell 2022, 82, 2335; 35714588 10.1016/j.molcel.2022.05.022

[4] E. Lundberg , G. H. H. Borner , Nat. Rev. Mol. Cell Biol. 2019, 20, 285. 30659282 10.1038/s41580-018-0094-y

[5] L. M. Smith , N. L. Kelleher , Nat. Methods 2013, 10, 186. 23443629 10.1038/nmeth.2369 PMC4114032

[6] J. P. McGee , P. Su , K. R. Durbin , M. A. R. Hollas , N. W. Bateman , G. L. Maxwell , T. P. Conrads , R. T. Fellers , R. D. Melani , J. M. Camarillo , J. O. Kafader , N. L. Kelleher , Nat. Commun. 2023, 14, 6478. 37838706 10.1038/s41467-023-42208-3 PMC10576781

[7] a) R. A. Lubeckyj , L. Sun , Mol Omics 2022, 18, 112; 34935839 10.1039/d1mo00335f PMC9066772

[8] a) M. Yang , H. Hu , P. Su , P. M. Thomas , J. M. Camarillo , J. B. Greer , B. P. Early , R. T. Fellers , N. L. Kelleher , J. Laskin , Angew. Chem., Int. Ed. 2022, 61, e202207371; 10.1002/anie.202200721 PMC9276647 35446460

[9] a) E. Berghmans , G. Van Raemdonck , K. Schildermans , H. Willems , K. Boonen , E. Maes , I. Mertens , P. Pauwels , G. Baggerman , Methods Protoc. 2019, 2, 44; 31164623 10.3390/mps2020044 PMC6632162

[10] J. M. Spraggins , D. G. Rizzo , J. L. Moore , M. J. Noto , E. P. Skaar , R. M. Caprioli , Proteomics 2016, 16, 1678. 27060368 10.1002/pmic.201600003 PMC5117945

[11] V. Delcourt , J. Franck , E. Leblanc , F. Narducci , Y. M. Robin , J. P. Gimeno , J. Quanico , M. Wisztorski , F. Kobeissy , J. F. Jacques , X. Roucou , M. Salzet , I. Fournier , EBioMedicine 2017, 21, 55. 28629911 10.1016/j.ebiom.2017.06.001 PMC5514399

[12] Y. Sun , Y. Liang , C. Wang , B. Zhao , Z. Liang , X. Zhang , Y. Zhang , L. Zhang , Anal. Chem. 2023, 95, 6846. 37074169 10.1021/acs.analchem.2c05401

[13] a) K. L. Schey , D. M. Anderson , K. L. Rose , Anal. Chem. 2013, 85, 6767; 23718750 10.1021/ac400832w PMC3749783

[14] Ed S. Lein , M J. Hawrylycz , N. Ao , M. Ayres , A. Bensinger , A. Bernard , A F. Boe , M S. Boguski , K S. Brockway , E J. Byrnes , L. Chen , Li Chen , T.‐M. Chen , M. Chi Chin , J. Chong , B E. Crook , A. Czaplinska , C N. Dang , S. Datta , N R. Dee , A L. Desaki , T. Desta , E. Diep , T A. Dolbeare , M J. Donelan , H.‐W. Dong , J G. Dougherty , B J. Duncan , A J. Ebbert , G. Eichele , et al., Nature 2007, 445, 168. 17151600 10.1038/nature05453

[15] D. Hu , X. Dong , Q. Wang , M. Liu , S. Luo , Z. Meng , Z. Feng , W. Zhou , W. Song , L.‐Q. Zhu , J. Alzheimer's Dis. 2023, 94, 737. 37302034 10.3233/JAD-230192

[16] C. Depp , T. Sun , A. O. Sasmita , L. Spieth , S. A. Berghoff , T. Nazarenko , K. Overhoff , A. A. Steixner‐Kumar , S. Subramanian , S. Arinrad , T. Ruhwedel , W. Möbius , S. Göbbels , G. Saher , H. B. Werner , A. Damkou , S. Zampar , O. Wirths , M. Thalmann , M. Simons , T. Saito , T. Saido , D. Krueger‐Burg , R. Kawaguchi , M. Willem , C. Haass , D. Geschwind , H. Ehrenreich , R. Stassart , K.‐A. Nave , Nature 2023, 618, 349. 37258678 10.1038/s41586-023-06120-6 PMC10247380

[17] S. C. Upadhya , A. N. Hegde , BMC Biochem. 2007, 8, S12. 18047736 10.1186/1471-2091-8-S1-S12 PMC2106363

[18] a) L. Harris , P. Rigo , T. Stiehl , Z. B. Gaber , S. H. L. Austin , M. D. M. Masdeu , A. Edwards , N. Urban , A. Marciniak‐Czochra , F. Guillemot , Cell Stem Cell 2021, 28, 863; 33581058 10.1016/j.stem.2021.01.003 PMC8110946

[19] T. Ashleigh , R. H. Swerdlow , M. F. Beal , Alzheimers Dement. 2023, 19, 333. 35522844 10.1002/alz.12683

[20] L. A. Ralat , V. Kalas , Z. Zheng , R. D. Goldman , T. R. Sosnick , W.‐J. Tang , J. Mol. Biol. 2011, 406, 454. 21185309 10.1016/j.jmb.2010.12.026 PMC3064465

[21] I. V. Kurochkin , E. Guarnera , I. N. Berezovsky , Trends Pharmacol. Sci. 2018, 39, 49. 29132916 10.1016/j.tips.2017.10.008

[22] a) J. Ma , T. Chen , S. Wu , C. Yang , M. Bai , K. Shu , K. Li , G. Zhang , Z. Jin , F. He , H. Hermjakob , Y. Zhu , Nucleic Acids Res. 2019, 47, D1211; 30252093 10.1093/nar/gky869 PMC6323926

