# Supplementary material for: High‐Throughput Proteoform Imaging for Revealing Spatial‐Resolved Changes in Brain Tissues Associated with Alzheimer's Disease
Source: Adv Sci (Weinh). 2025 Mar 12;12(17):2416722. doi: 10.1002/advs.202416722 (PMC12061265; doi:10.1002/advs.202416722)
Supplement: Supplementary file 1 — Supporting Information [file ADVS-12-2416722-s001.pdf]

ADVANCED SCIENCE
Open Access

Supporting Information

for *Adv. Sci.*, DOI 10.1002/advs.202416722

High-Throughput Proteoform Imaging for Revealing Spatial-Resolved Changes in Brain Tissues Associated with Alzheimer's Disease

Yue Sun, Dan Liu, Yu Liang, Xue Yang, Xinxin Liu, Baofeng Zhao, Zhen Liang, Yukui Zhang and Lihua Zhang**

Supporting Information

High-Throughput Proteoform Imaging for Revealing Spatial-Resolved Changes in Brain Tissues Associated with Alzheimer's Disease

Yue Sun,# Dan Liu,# Yu Liang, Xue Yang, Xinxin Liu, Baofeng Zhao, Zhen Liang, Yukui Zhang, and Lihua Zhang**

\# These authors contributed equally to this work.

* Corresponding authors

E-mail: yuliang@dicp.ac.cn; lihuazhang@dicp.ac.cn

Table of Contents

Figure S1. Total ion of chromatography of four standard proteins (100 pg) using the 50 μm i.d TEOOS-BTMSPA monolith……S2

Figure S2. Region-specific top-down LC-MS/MS analysis of LCM-derived mouse brain tissue sections……S3

Figure S3. Immunofluorescence image of Pcp4 protein……S4

Figure S4. Differential detection of proteoforms in the different brain regions of WT and 5×FAD mice……S5

Figure S5. Differential MSI-generated proteoforms across WT and AD mouse brains……S6

Figure S6. MS images of Aβ proteoforms at the spatial resolution of 20 μm……S7

Table S1. The intensity of extracted ions from the base peak chromatogram……S8

Table S2. Proteoform identification in tissue sections with different areas……S9

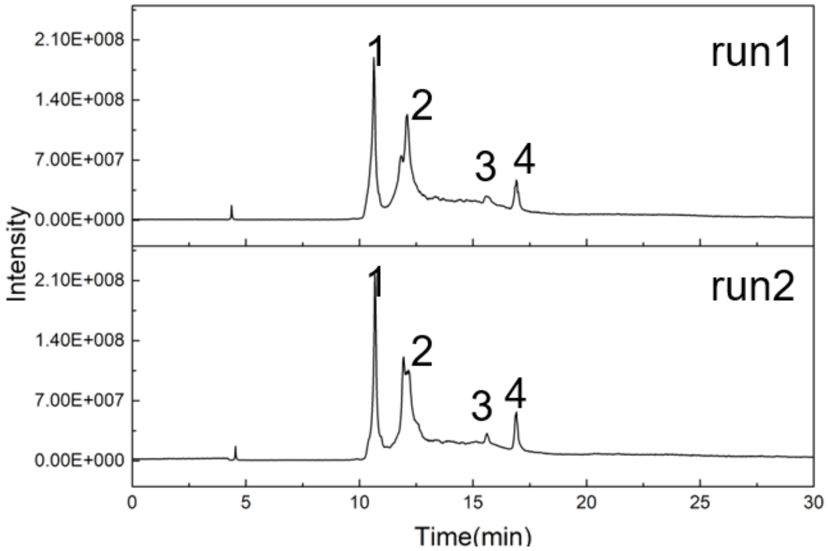

Figure S1. Total ion of chromatography of four standard proteins (100 pg) using the 50 µm i.d. TEOOS-BTMSPA monolith. (1) Ribonuclease A (7.3 fmol), (2) Cytochrome c (7.7 fmol), (3) Myoglobin (6.0 fmol), (4) Carbonic anhydrase (3.3 fmol)

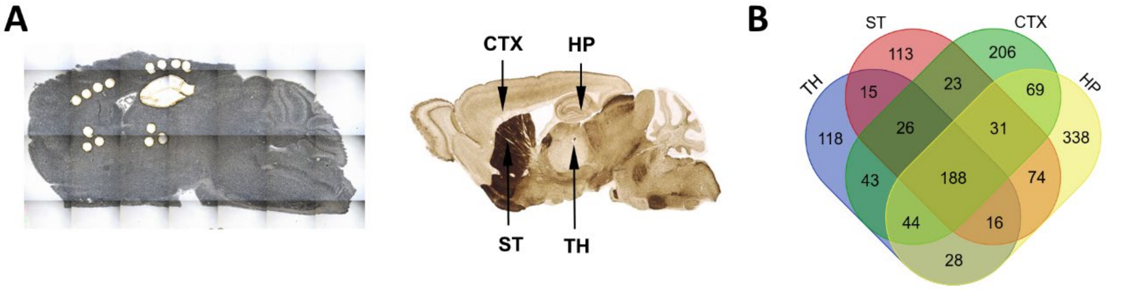

Figure S2. Region-specific top-down LC-MS/MS analysis of LCM-derived mouse brain tissue sections. (A) Images of the tissue section after sectioning with LCM from the CTX, HP, TH, and ST regions as labeled. CTX was separated into two parts due to its large range, and HP is segmented according to its subregions. (B) Top-down identification results of proteoforms in four regions.

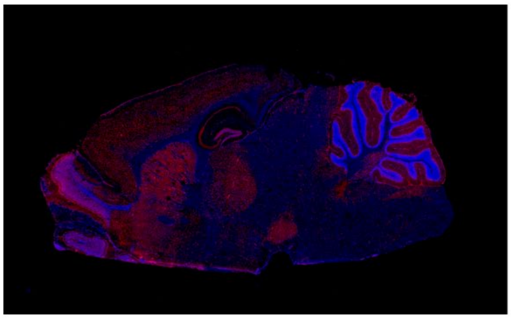

Figure S3. Immunofluorescence image of Pcp4 protein.

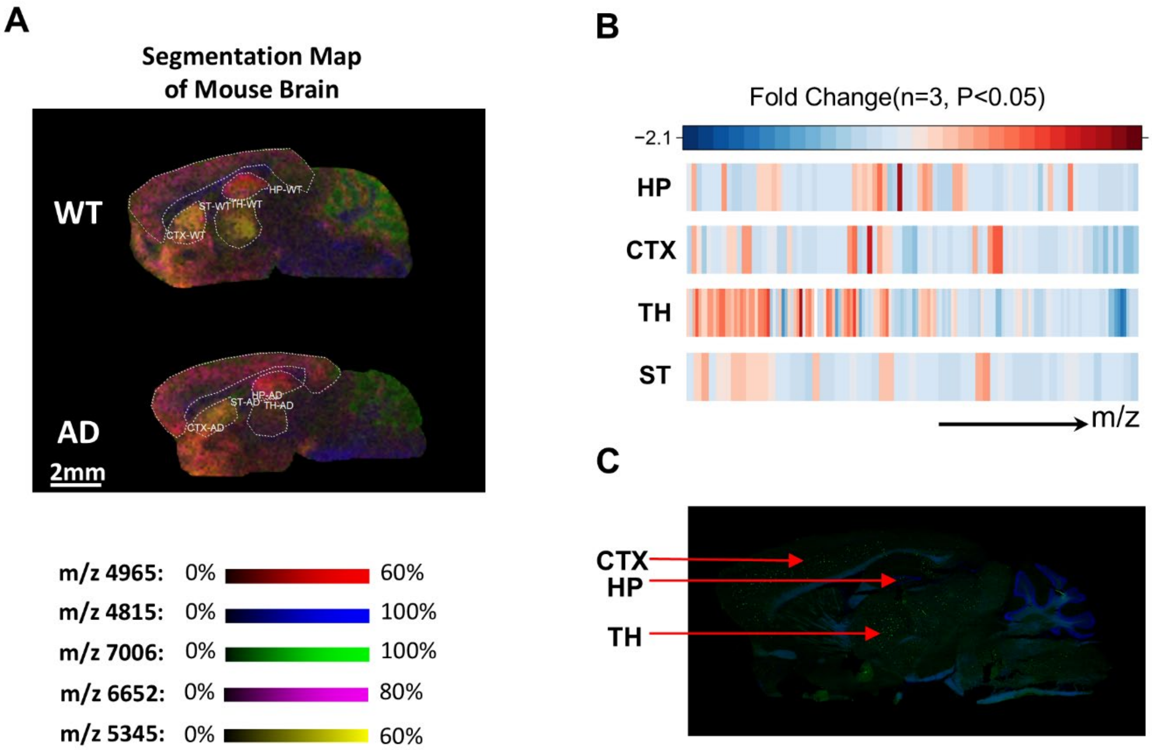

Figure S4. Differential detection of proteoforms in the different brain regions of WT and 5×FAD mice. (A) Segmentation map of WT and AD mouse brain. (B) Quantitation for the differentially detected proteoforms (AD/WT) in HP, CTX, TH and ST regions. N=3, $P < 0.05$, unpaired two-tailed t test. (C) Localization of Aβ aggregates as indicated by ThS staining in the AD brain tissue section.

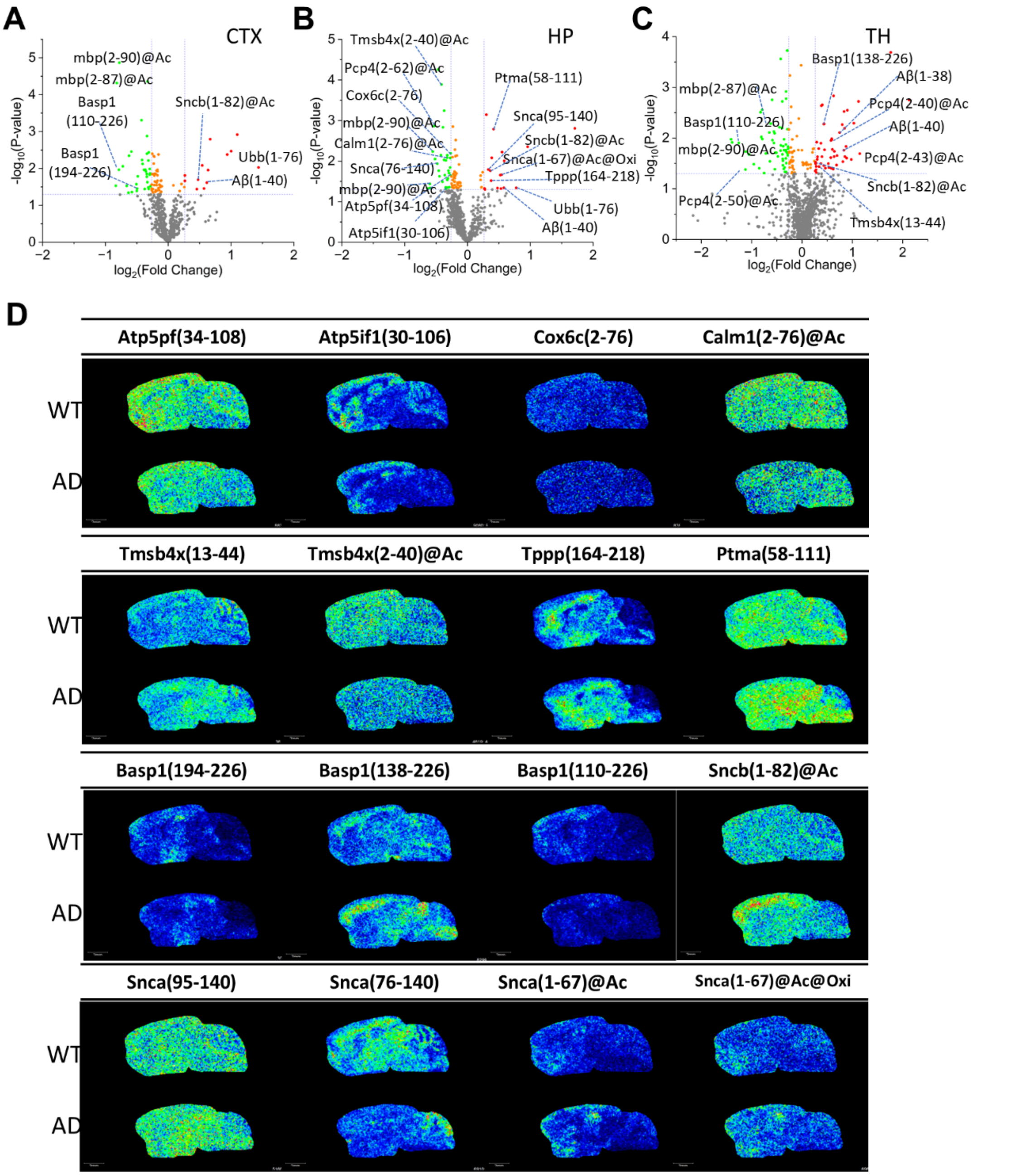

Figure S5. Differential MSI-generated proteoforms across WT and AD mouse brains. Volcano plots generated from label free quantitation using MSI dataset of CTX (A), HP (B) and TH (C) regions from WT and AD samples. (D) MS images of quantitative proteoforms with significant differences between WT and AD mouse brain (supplemental data to Figure 2C). N=3, P < 0.05, unpaired two-tailed t test.

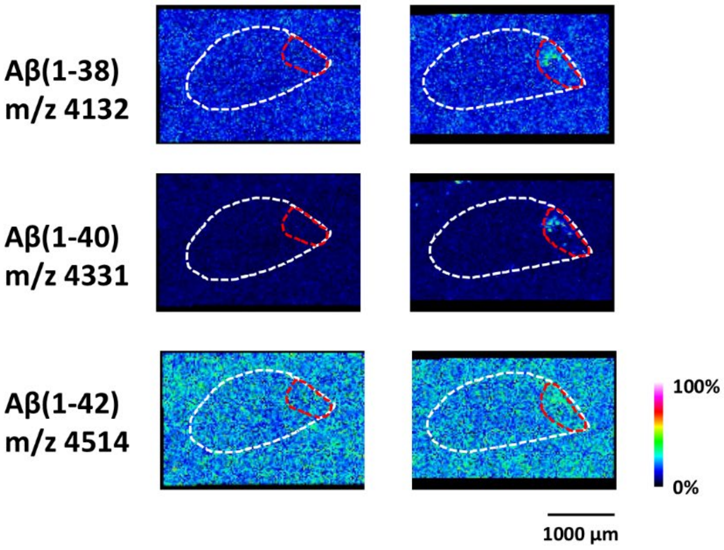

Figure S6. MS images of Aβ proteoforms at the spatial resolution of 20 µm.

Table S1. The intensity of extracted ions from the base peak chromatogram using TEOOS-BTMSPA monoliths with 100 μm i.d. and 50 μm i.d., respectively

		**0.5mm^2**		**0.1mm^2**	
**m/z**	**z**	**100μm**	**50μm**	**100μm**	**50μm**
828.0926	6	3.59E+08	1.90E+09	8.59E+07	4.81E+08
951.356	17	3.78E+06	1.20E+07	1.70E+05	1.09E+06
909.6357	19	1.74E+06	8.07E+06	8.40E+04	4.87E+05
1224.529	7	3.18E+07	1.15E+08	4.54E+06	3.08E+07
1278.803	7	1.13E+07	7.42E+07	3.40E+05	5.81E+06
837.3824	13	3.19E+07	1.29E+08	7.66E+05	1.52E+07
1085.082	13	9.65E+06	1.03E+08	1.52E+05	3.83E+06
907.9832	6	4.75E+06	1.23E+07	4.19E+05	2.01E+06
Average		5.67E+07	2.94E+08	1.15E+07	6.75E+07

Table S2. Proteoform identification in tissue sections with different areas using TEOOS-BTMSPA monoliths with 100 μm i.d. and 50 μm i.d., respectively.

**Section area**	**0.5mm^2**		**0.1mm^2**	
Column i.d.	**100μm**	**50μm**	**100μm**	**50μm**
01	210	343	98	236
02	226	406	99	210
Average	218	374	98	223
