# Supplementary material for: High‐Throughput Proteoform Imaging for Revealing Spatial‐Resolved Changes in Brain Tissues Associated with Alzheimer's Disease
Source: Adv Sci (Weinh). 2025 Mar 12;12(17):2416722. doi: 10.1002/advs.202416722 (PMC12061265; doi:10.1002/advs.202416722)
Supplement: Supplementary file 3 — Supplemental Table S4 [file ADVS-12-2416722-s002.pdf]

ADVANCED SCIENCE
Open Access

# Supporting Information

for *Adv. Sci.*, DOI 10.1002/advs.202416722

## High-Throughput Proteoform Imaging for Revealing Spatial-Resolved Changes in Brain Tissues Associated with Alzheimer's Disease

*Yue Sun, Dan Liu, Yu Liang*, Xue Yang, Xinxin Liu, Baofeng Zhao, Zhen Liang, Yukui Zhang and Lihua Zhang**

**Supplemental Table S4**. Proteoform images with annotation.

| Basp1(194–226) | Snap91(868–896) | Basp1(193–226) |
|---|---|---|
| 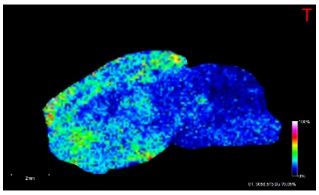 | 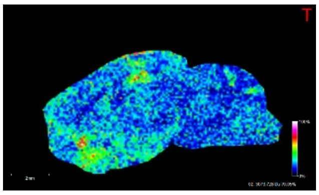 | 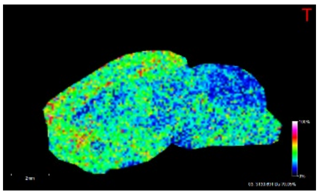 |
| Prrt2(2–36)@Ac | Basp1(187–226) | Basp1(112–153) |
| 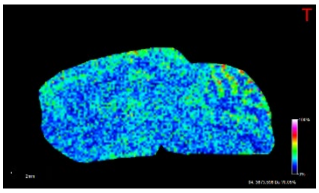 | 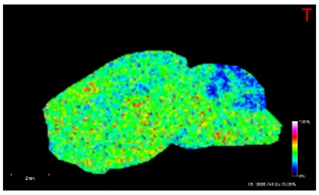 | 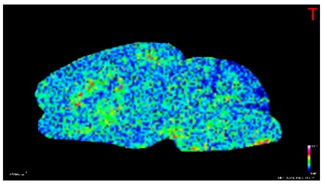 |
| Lrrk2(111–144)@[–180.91Da] | Ptma(2–36)@Ac | Basp1(183–226)@[–215.86Da] |
| 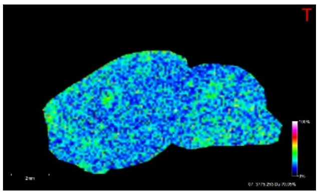 | 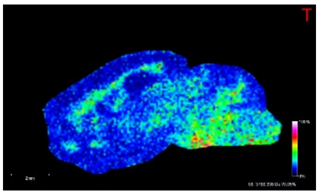 | 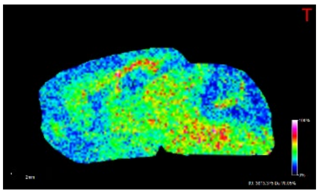 |
| Tmsb4x(2–34)@Ac | Basp1(110–153) | Tmsb4x(2–35)@Ac |
| 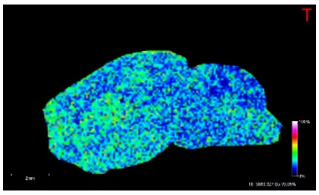 | 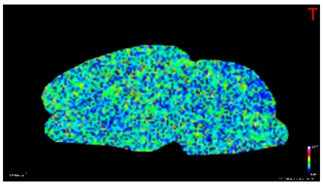 | 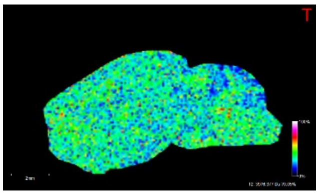 |
| Tmsb4x(2–35)@Ac | Aβ(1–38) | Tmsb4x(9–44) |
| 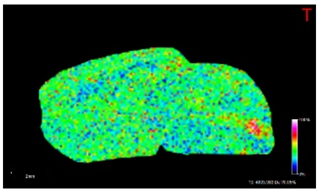 | 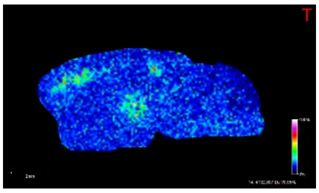 | 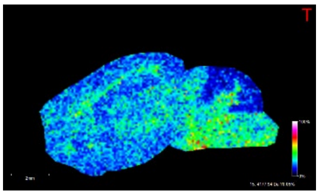 |
| Calm3(1–39)@[–273.2Da] | Calm3(1–39)@[–239.22Da] | Calm3(1–39)@[–223.22Da] |
| 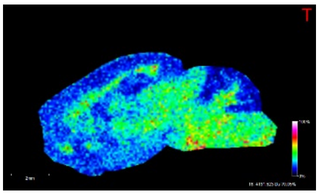 | 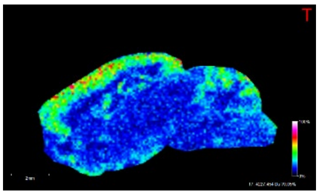 | 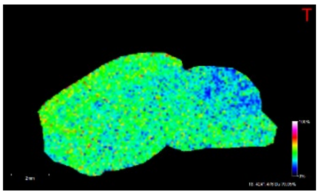 |
| Tmsb4x(8–44) | Mbp(2–38)@Ac | Pcp4(2–40)@Ac |

| | | |
|---|---|---|
| 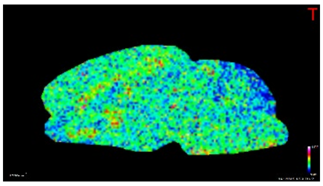 | 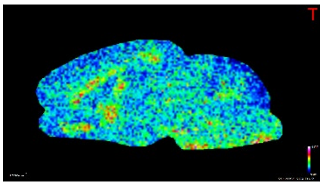 | 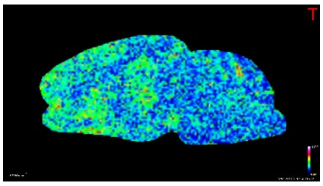 |
| **Ppp1r1b(1–36)@Ac** | **Aβ(1–40)** | **Sncb(1–41)@Ac** |
| 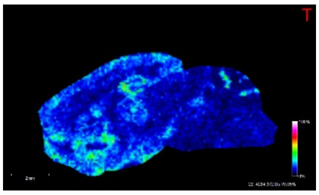 | 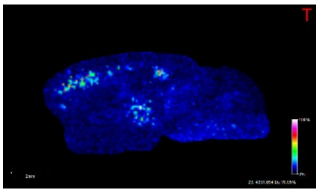 | 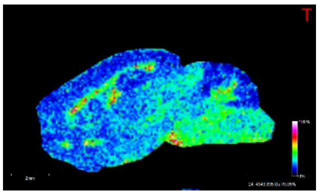 |
| **Tmsb4x(2–38)@Ac** | **Zfp444(72–112)@[+62.14Da]** | **Rnf217(355–392)@[–177.11Da]** |
| 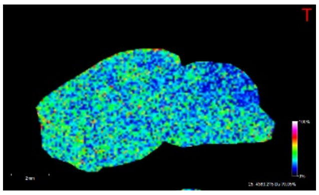 | 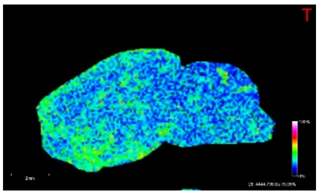 | 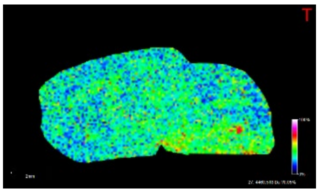 |
| **Pcp4(2–43)@Ac** | **Tmsb4x(2–40)@Ac** | **Basp1(174–226)@[–288.99Da]** |
| 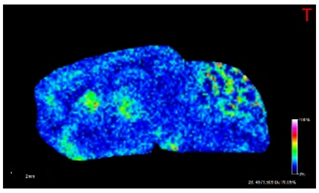 | 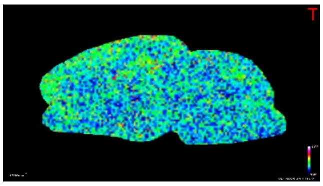 | 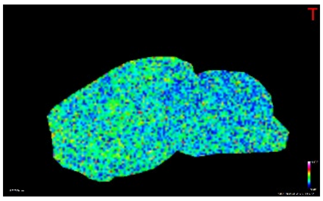 |
| **Calm3(1–39)@Ac** | **Syn1(605–651)** | **Tmsb4x(2–41)@Ac** |
| 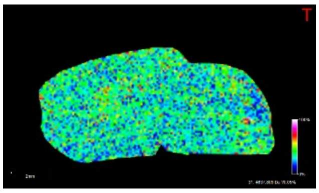 | 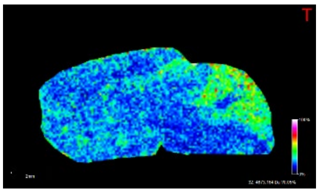 | 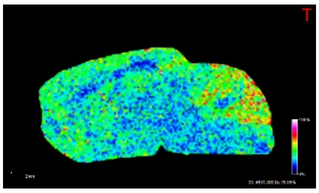 |
| **Tmsb4x(2–42)@Ac** | **Pcp4(21–62)** | **Mbp(2–43)@Ac** |
| 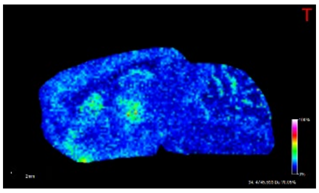 | 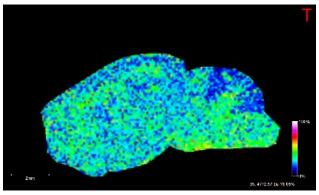 | 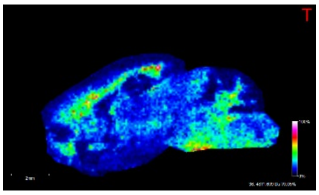 |
| **Sncb(92–133** | **Tmsb4x(2–49)@Ac** | **Cox8a(26–69)** |
| 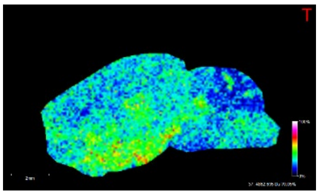 | 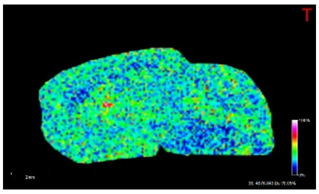 | 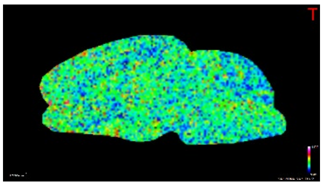 |
| **Tmsb4x(2–44)@Ac@[–** | **Basp1(174–226)** | **Tmsb10(2–44)@Ac** |

| | | |
|---|---|---|
| 57.02Da] | | |
| 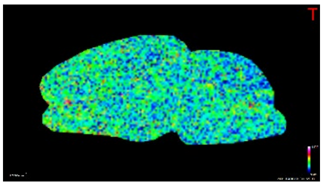 | 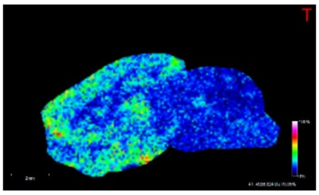 | 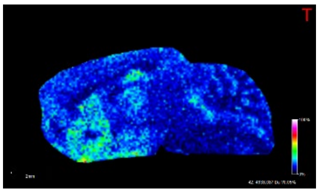 |
| Tmsb4x(2–44)@Ac | Tmsb4x(2–50)@Phospho | Ptms(53–97)@[–199.48Da] (2–44)@Ac@[+48.04Da] |
| 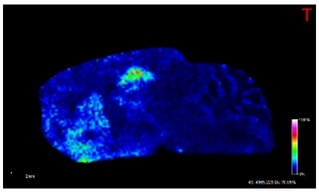 | 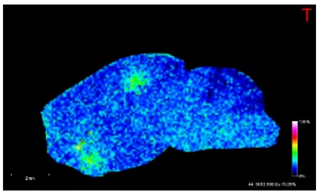 | 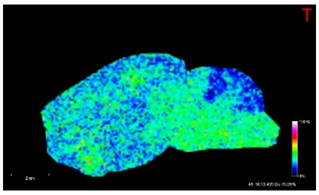 |
| Ptms(53–97)@[–199.48Da] | Tmsb4x(2–44)@Ac@Phospho | Dpy30(2–47) |
| 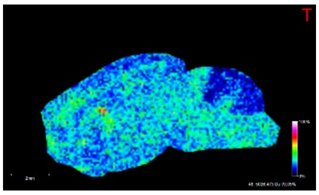 | 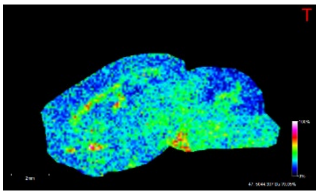 | 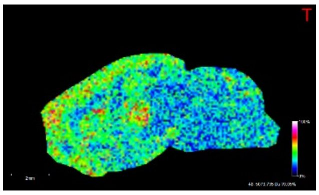 |
| Tmsb4x(2–44)@Ac@[+139.21Da] | H3c1(89–136)@[–269.12Da] | Tmsb4x(1–50)@Phospho |
| 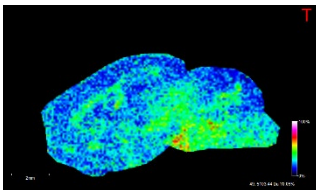 | 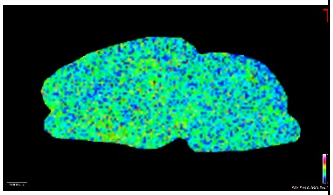 | 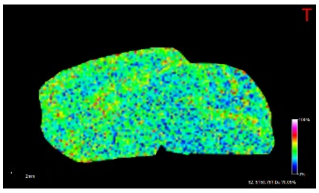 |
| Cplx2(89–134)@[+57.91Da] | Tmsb4x(1–50)@Ac@Phospho | Pcp4(2–49)@Ac |
| 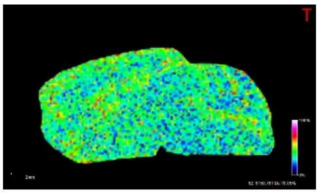 | 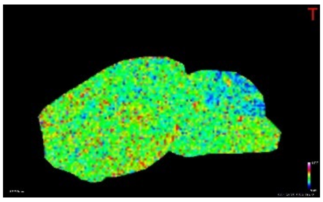 | 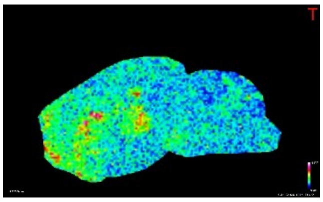 |
| Pcp4(2–50)@Ac | Basp1(169–226) | Hspe1(2–57)@Ac@[–422.65Da] |
| 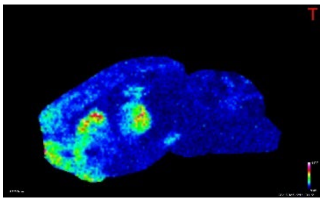 | 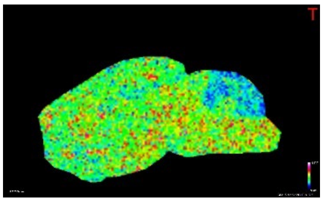 | 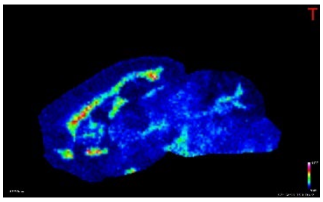 |
| Nrgn(1–52)@Ac | Tmsb4x(1–44)@[+451.19Da] | Snca(91–140) |

| | | |
|---|---|---|
| 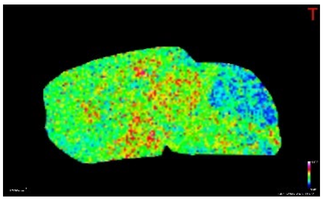 | 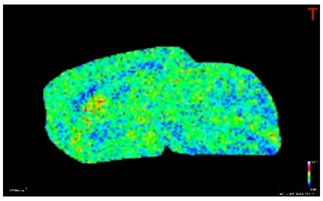 | 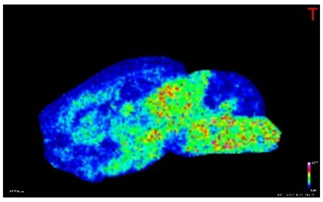 |
| Ptms(52–99) | Cplx2(84–134) | Calm1(99–149)@[–157.98Da] |
| 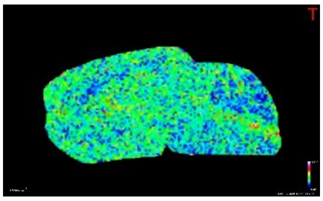 | 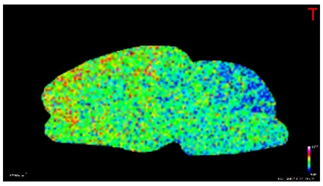 | 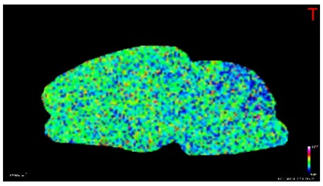 |
| Atp5f1e(2–52) | Cplx2(1–50)@Ac | Mbp(2–53)@Ac |
| 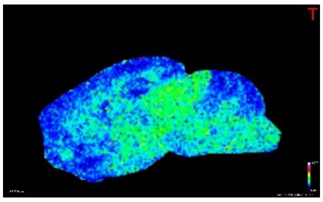 | 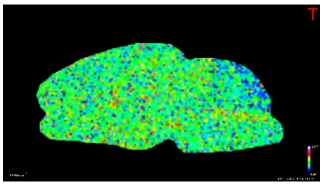 | 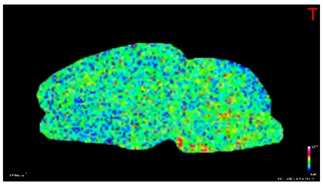 |
| Tmsb4x(1–50)@Ac*2@Phospho*2 | Sncb(83–133) | Tppp(164–218) |
| 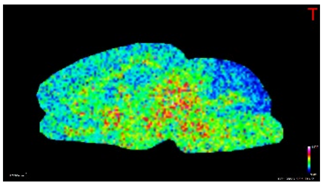 | 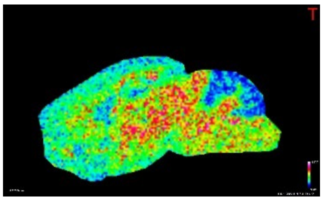 | 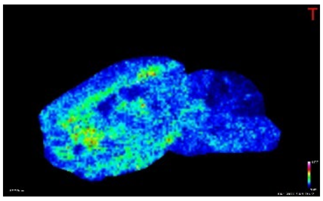 |
| Basp1(162–226) | Hnrnpu(2–56)@Ac | Ptma(58–111)@[+22.88Da] |
| 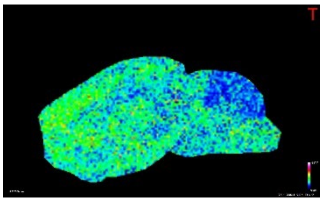 | 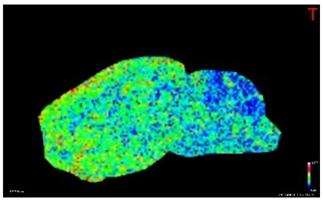 | 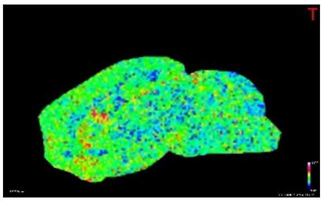 |
| Calm1(94–149)@[–115.06Da] | Uba52(77–128)@Ac | Cox7b(25–80) |
| 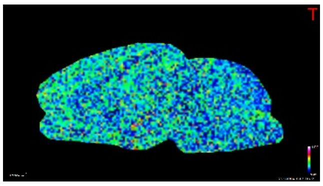 | 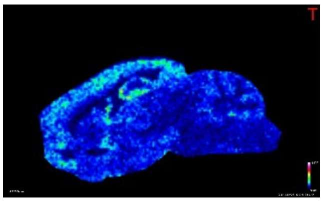 | 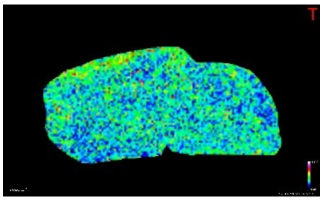 |
| Cox7b(25–80)@Oxi | H3c2(79–136)@[–113.12Da] | Cox7a2(24–83) |

| | | |
|---|---|---|
| 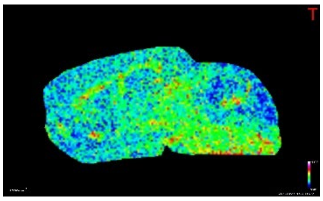 | 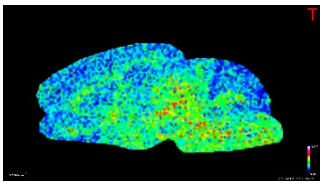 | 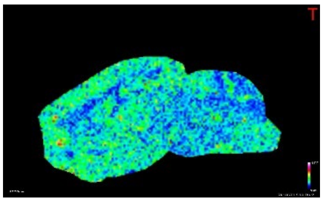 |
| **Fau(75–133)** | **Sncb(1–64)@Ac** | **Basp1(155–226)** |
| 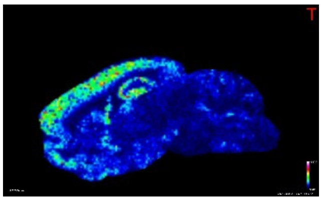 | 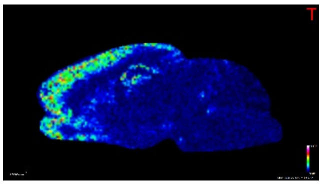 | 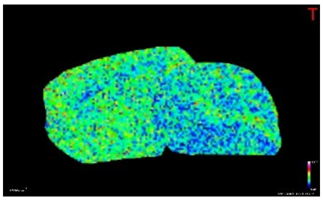 |
| **Pcp4(2–62)@Ac** | **Basp1(154–226)** | **H3c2(76136)@[–101.19Da]** |
| 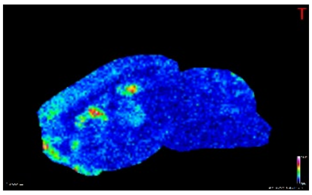 | 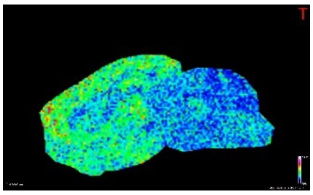 | 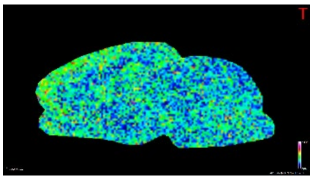 |
| **Pcp4(2–62)@Ac@Phospho** | **Pcp4(1–62)** | **Basp1(153–226)** |
| 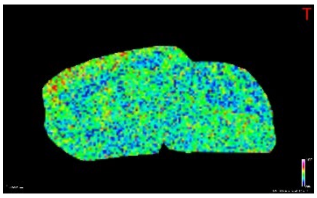 | 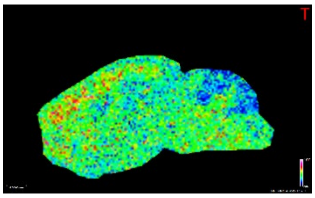 | 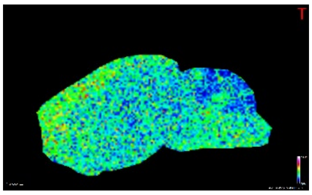 |
| **Sncb(1–66)@Ac** | **H2bc14(64–126)@[–73.2Da]** | **Snca(76–140)** |
| 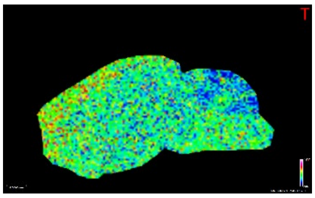 | 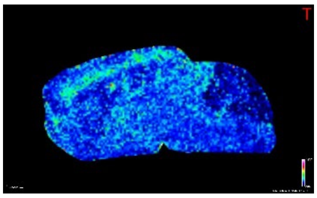 | 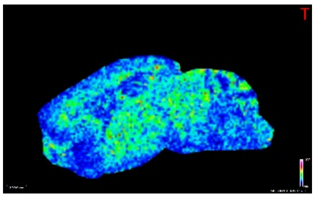 |
| **Snca(1–67)@Ac** | **Mt3(1–68)** | **Basp1(149–226)@[–130.03Da]** |
| 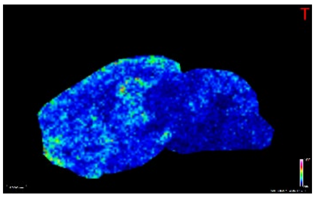 | 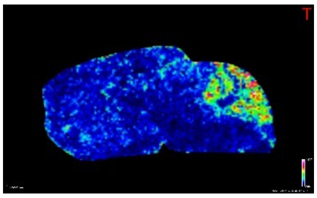 | 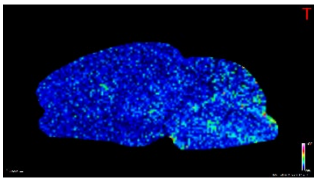 |
| **Rida(71–135)** | **Sst(25–87)** | **Cplx1(162)@Ac** |

| | | |
|---|---|---|
| 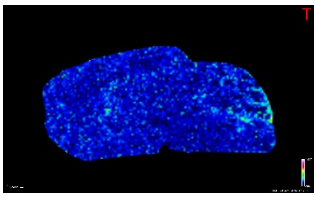 | 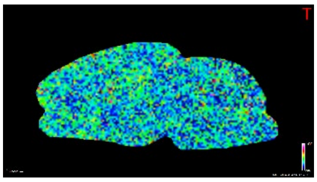 | 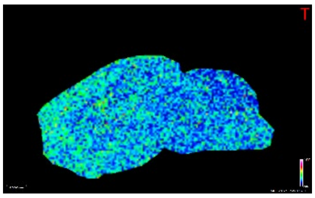 |
| **Ncam1(1018–1089)** | **Atox1(2–68)** | **Prrt2(2–72)@Ac** |
| 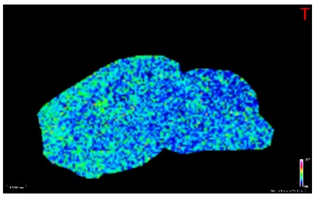 | 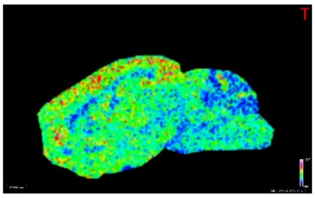 | 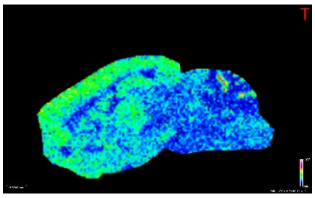 |
| **Pcp4l1(2–66)@Ac** | **Cplx2(68–134)@[–226.02Da]** | **Pcp4l1(2–66)@Ac@Phospho** |
| 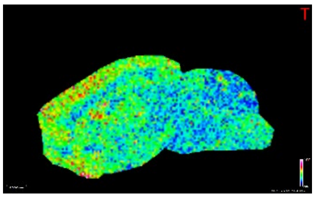 | 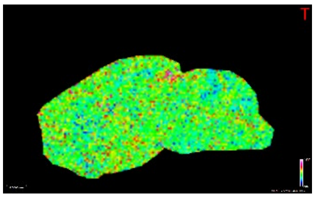 | 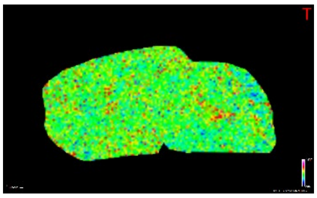 |
| **Rplp2(1–72)** | **Nrgn(1–76)@Ac** | **Mbp(2–67)@Ac** |
| 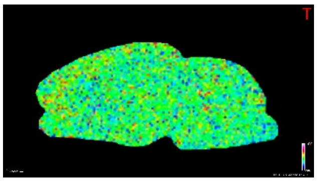 | 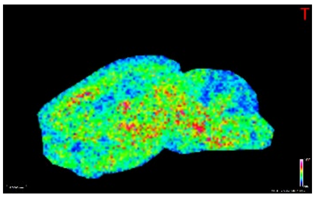 | 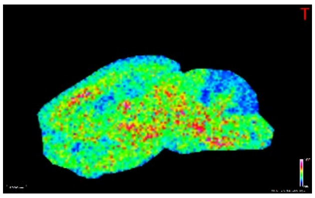 |
| **Pcp4l1(2–68)@Ac** | **Cend1(1–73)@Ac** | **H2ax(2–72)@Ac** |
| 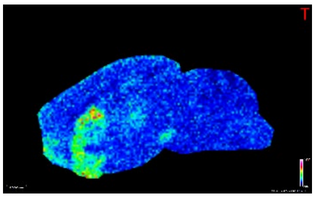 | 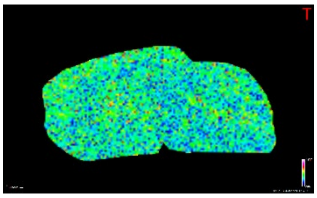 | 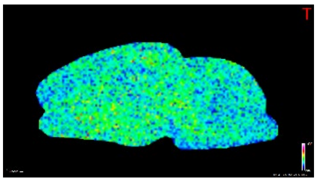 |
| **Nrgn(1–78)@Ac** | **Nrgn(1–78)@Ac@Methyl** | **Nrgn(1–78)@Ac@Oxi** |
| 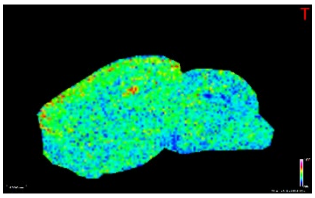 | 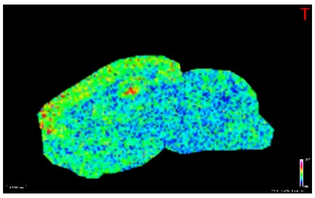 | 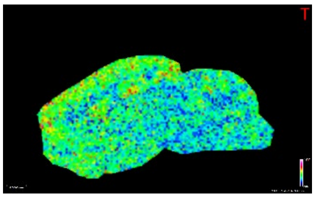 |
| **Basp1(110–193)** | **Snca(68–140)** | **Nrgn(1–78)@Ac@[+54.92Da]** |
| 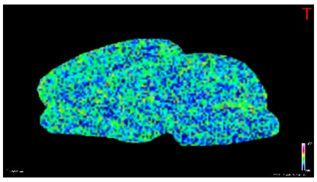 | 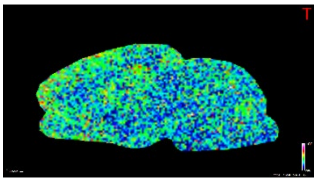 | 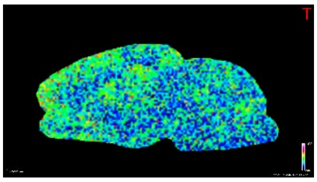 |

| | | |
|---|---|---|
| **Nrgn(1–78)@Ac@Methyl@Phospho** | **Atp5mc2(72–146)@Trimethyl** | **Gng2(2–71)@Ac@[–10.94Da]** |
| 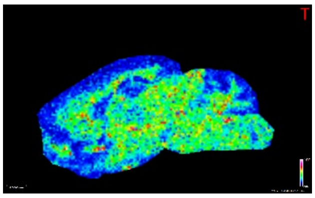 | 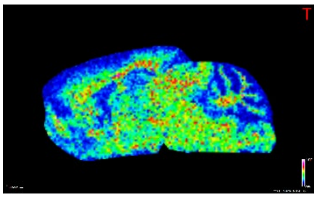 | 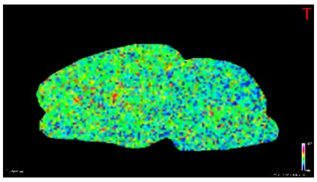 |
| **Nrgn(1–78)@Ac@[+237.22Da]** | **Hspe1(27–102)@[–250.47Da]** | **Basp1(143–226)@[–1Da]** |
| 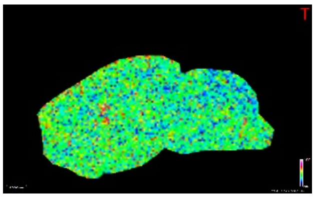 | 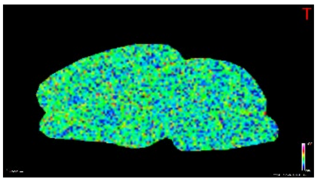 | 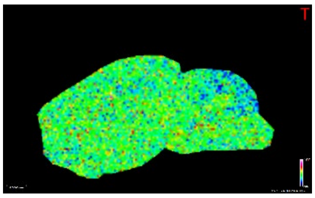 |
| **ATP8(1–67)** | **Calm1(82–149)@[+20.36Da]** | **Sncb(1–78)@Ac** |
| 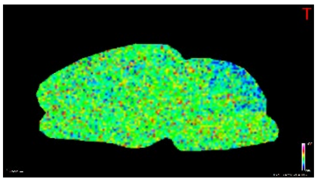 | 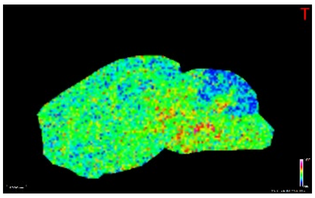 | 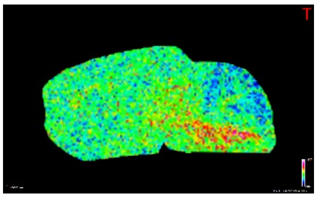 |
| **Rps28(1–69)@Ac** | **Uqcrfs1(1–78)** | **Mbp(2–72)@Ac** |
| 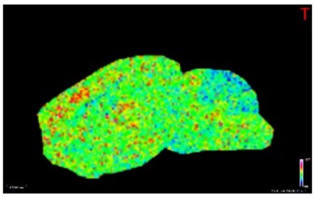 | 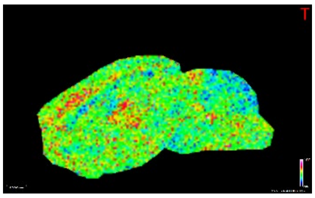 | 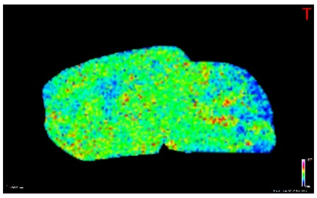 |
| **Sod1(2–80)@Ac@[–418.65Da]** | **Uqcrfs1(1–78)@Ac** | **Uqcrfs1(1–78)@Ac@Oxi** |
| 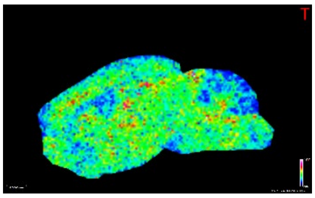 | 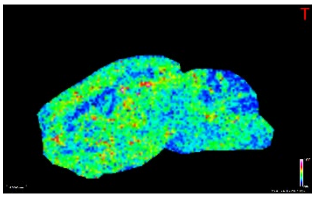 | 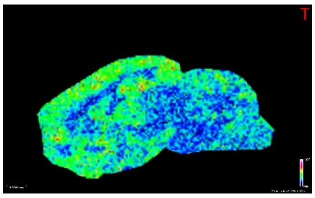 |
| **Calm1(82–149)@[+140.17Da]** | **Ndufv3(36–104)** | **Ndufv3(36–104)@Ac** |

| | | |
|---|---|---|
| 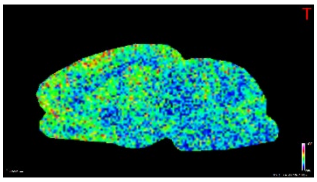 | 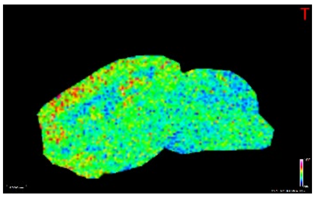 | 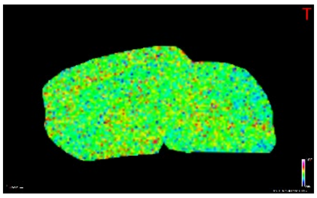 |
| Rpl38(2–70) | Atp5me(2–71) | Ndufa1(1–70) |
| 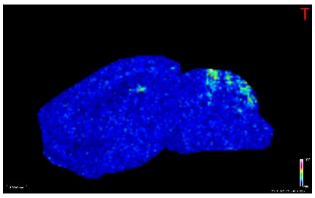 | 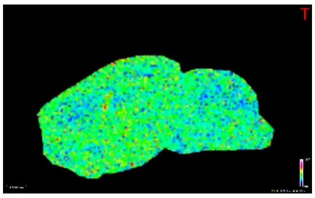 | 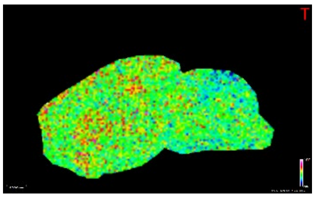 |
| Atp5me(2–71)@Ac | Sncb(1–82)@Ac | Camk2a(129–200) |
| 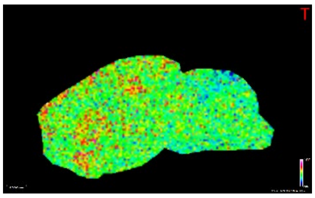 | 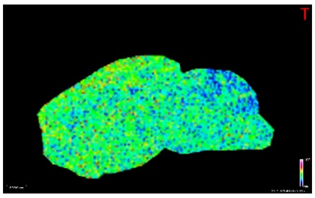 | 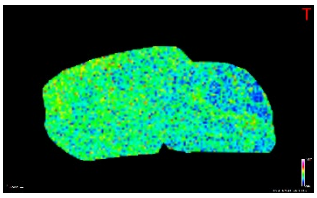 |
| Hopx(2–73)@Ac | Atp5me(1–71) | H2bc14(51–126) |
| 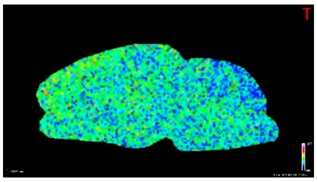 | 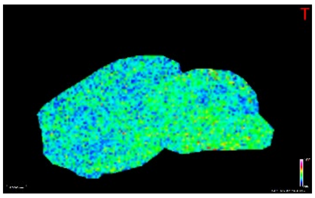 | 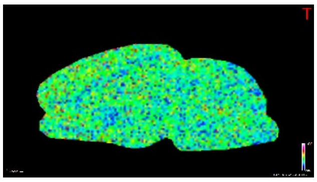 |
| Basp1(138–226) | Cox6c(2–76) | Basp1(137–226) |
| 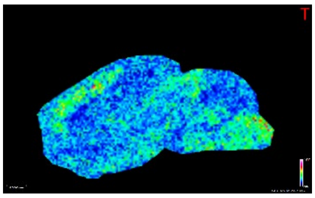 | 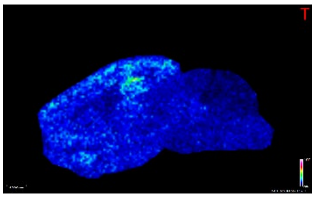 | 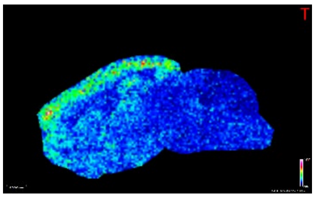 |
| Cox6c(2–76)@Ac | Calm1(2–76)@Ac@[+38.76Da] | Calm1(2–76)@Ac@[+48.64Da] |
| 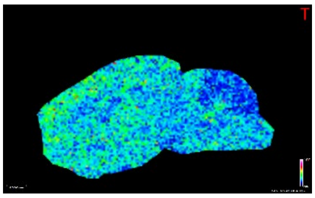 | 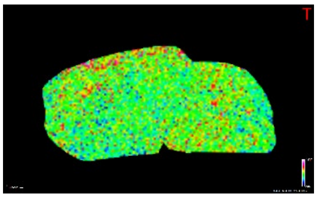 | 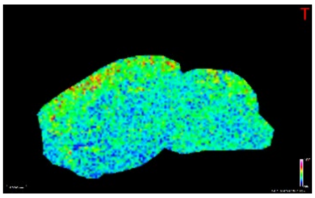 |
| Calm1(2–75)@Ac@[+181.85Da] | Calm1(76–149)@[–115.33Da] | Ubb(1–74) |

| | | |
|---|---|---|
| 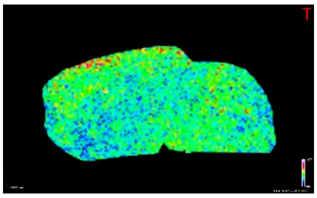 | 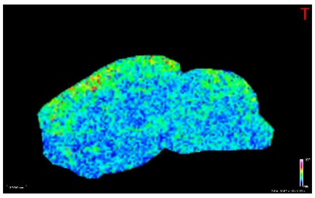 | 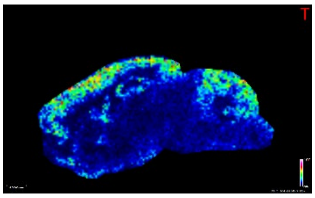 |
| **Calm1(2–76)@Ac@[+155.16Da]** | **Ubb(1–76)** | **Ubb(1–76)@Ac** |
| 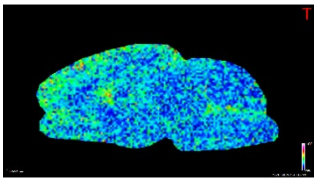 | 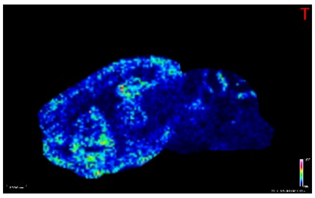 | 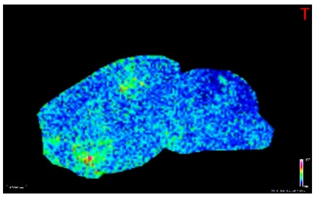 |
| **Basp1(134–226)** | **Atp6v1g2(1–77)@[–228.44Da]** | **Ufm1(2–83)** |
| 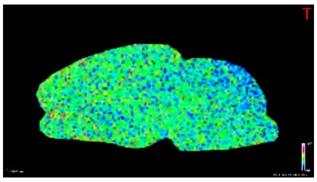 | 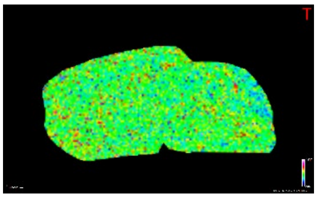 | 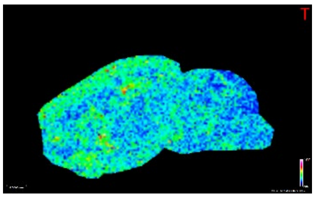 |
| **Atp5pf(34–108)** | **Ufm1(2–83)@Ac** | **Basp1(131–226)** |
| 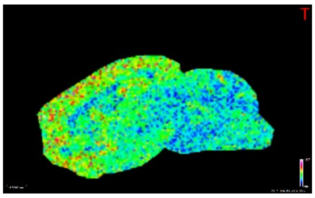 | 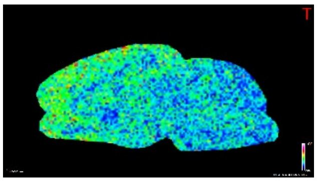 | 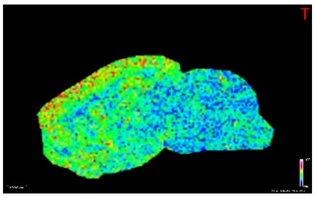 |
| **Atp6v1g2(2–77)** | **Atp6v1g2(2–77)@Ac** | **Atp5pf(33–108)** |
| 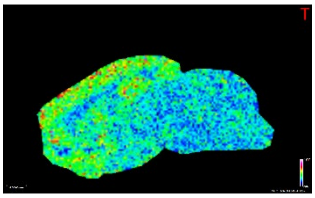 | 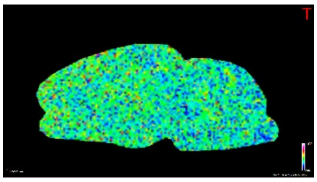 | 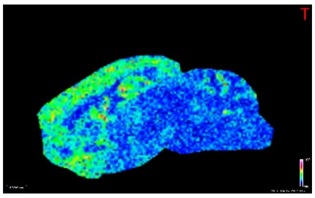 |
| **Atp5pf(33–108)@Oxi** | **Uqcrh(14–89)** | **Mbp(44–126)** |
| 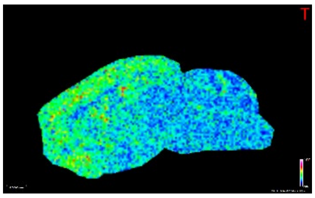 | 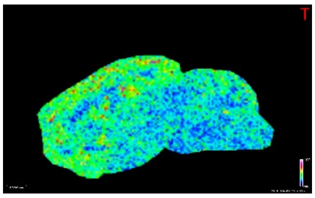 | 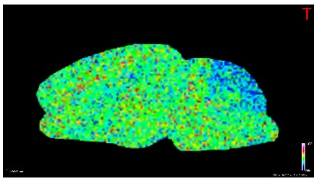 |
| **Uqcrh(14–89)@Ac** | **Mbp(44–126)@Methyl** | **Atp5if1(30–106)** |

| | | |
|---|---|---|
| 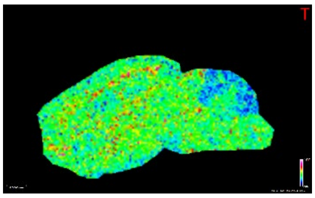 | 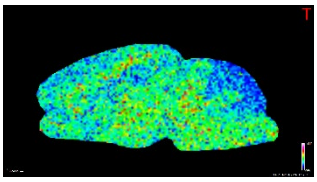 | 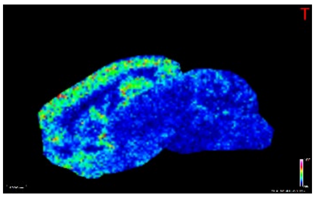 |
| **Tsc22d3(1–80)@Ac** | **Ptma(29–111)@Ac** | **Spata33(1–86)@[–218.95Da]** |
| 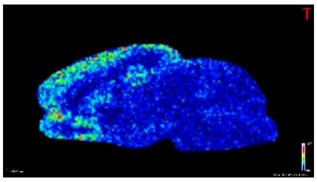 | 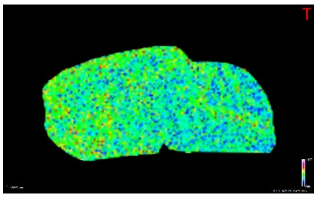 | 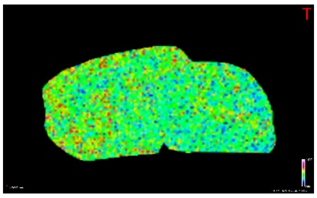 |
| **Atp5if1(29–106)** | **Sncb(1–91)@Ac** | **Ndufa4(1–82)@[–5.81Da]** |
| 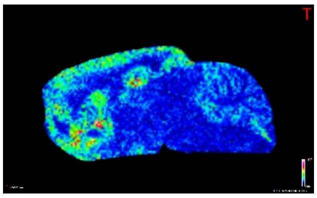 | 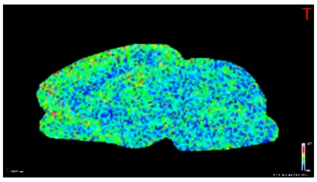 | 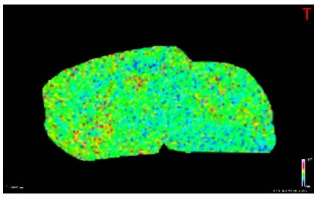 |
| **Mbp(44–128)** | **Snca(1–94)@Ac** | **Tsc22d1(1–86)@Ac** |
| 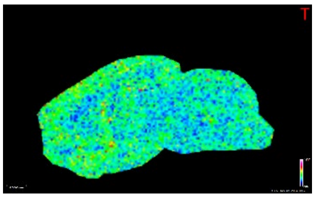 | 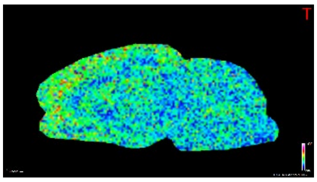 | 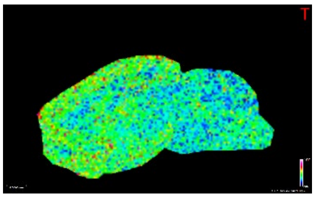 |
| **Basp1(125–226)** | **Tsc22d1(1–86)@[+58.03Da]** | **Trmt11(2–82)@Ac** |
| 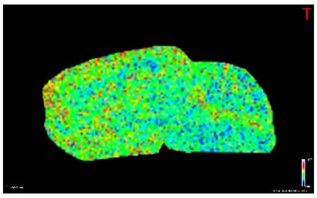 | 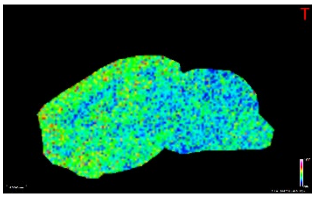 | 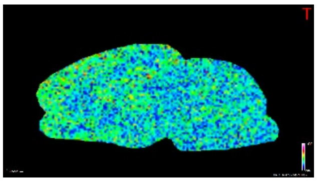 |
| **Tsc22d1(1–86)@[+74.02Da]** | **Cplx1(1–81)@Ac** | **Basp1(1–89)@[+79.17Da]** |
| 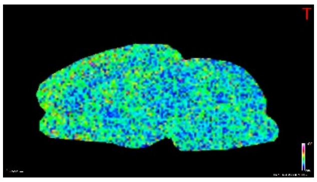 | 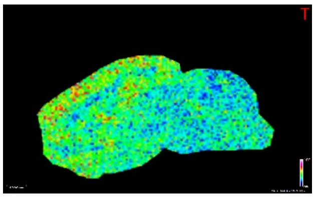 | 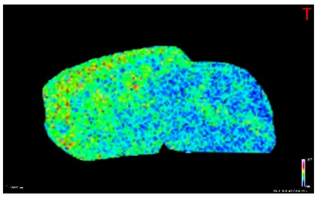 |
| **Tsc22d1(1–86)@Ac@[+55.27Da]** | **H2az1(1–90)@Ac** | **Mbp(2–86)@Ac** |

| | | |
|---|---|---|
| 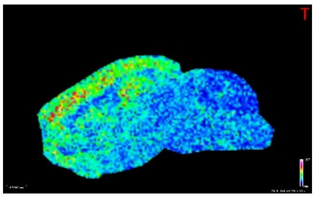 | 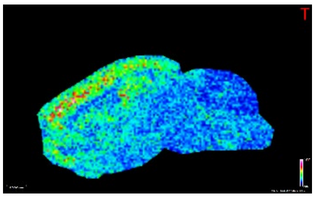 | 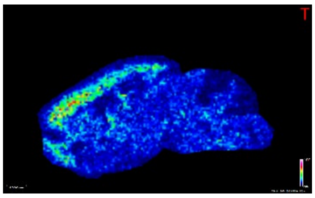 |
| **Krtap17–1(2–109)@Ac@[+38.64Da]** | **Cox6a1(27–111)** | **Cplx1(1–83)@Ac** |
| 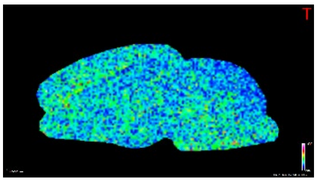 | 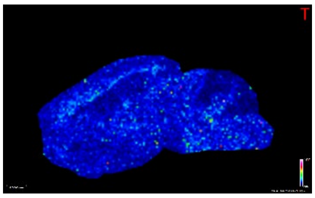 | 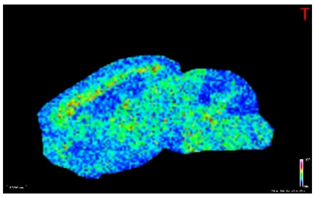 |
| **Mbp(2–87)@Ac** | **Mbp(2–87)@Ac@Phospho** | **Cplx2(1–83)@Ac** |
| 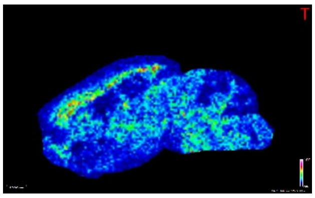 | 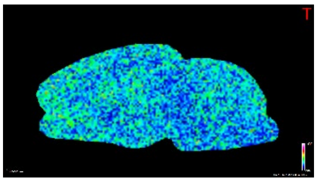 | 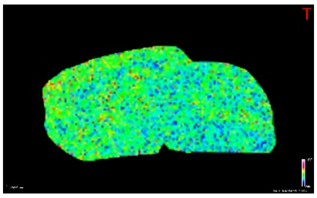 |
| **Dbi(2–87)@Ac** | **Dbi(2–87)@Ac@Oxi** | **Mbp(2–89)@Ac** |
| 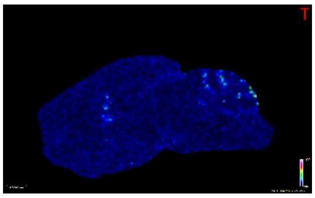 | 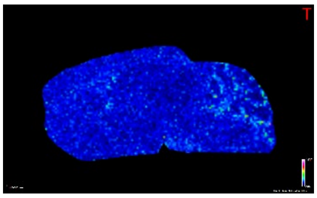 | 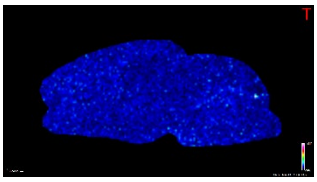 |
| **Basp1(119–226)** | **Cox6b1(2–86)@Ac** | **Mbp(2–90)@Ac** |
| 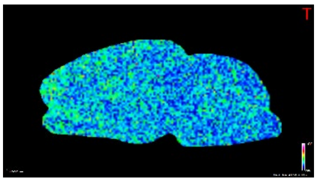 | 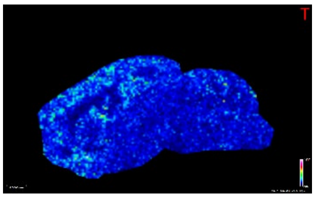 | 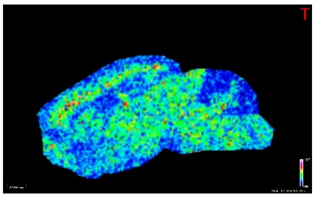 |
| **Mbp(2–90)@Ac@Phospho** | **Dbi(2–87)@Ac@[+221.21Da]** | **Dynll2(2–89)@Ac** |
| 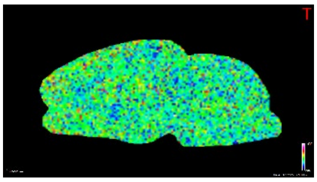 | 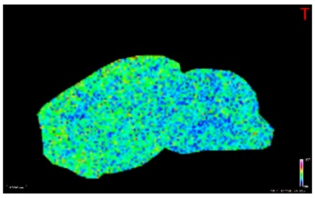 | 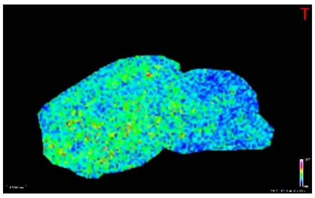 |
| **Dynll1(2–89)@Ac** | **Mbp(2–93)@Ac** | **Sh3bgrl3(2–93)@Ac** |

| | | |
|---|---|---|
| 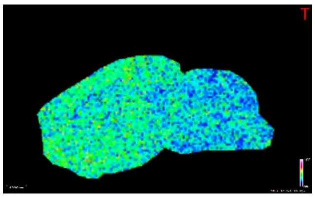 | 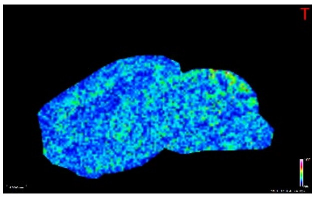 | 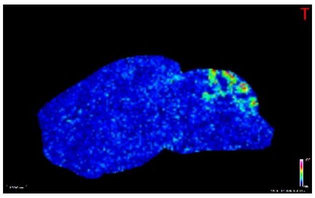 |
| **S100a1(2–94)@Ac** | **Basp1(112–226)** | **Sumo2(2–93)@Ac** |
| 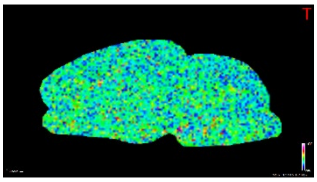 | 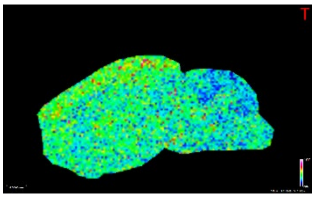 | 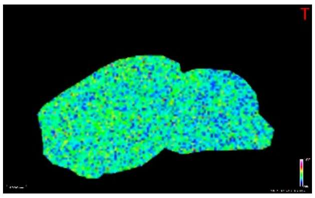 |
| **Calm1(2–94)@Ac** | **Mbp(2–95)@Ac** | **Basp1(110–226)** |
| 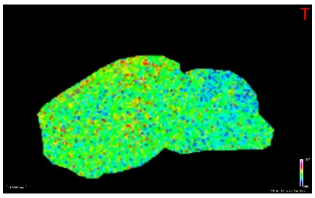 | 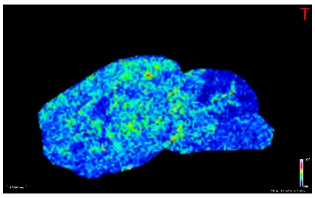 | 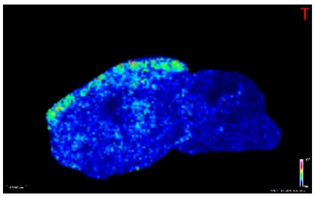 |
| **Cox5b(31–128)** | **Cplx2(1–92)@Ac** | **Cox5b(31–128)@Ac** |
| 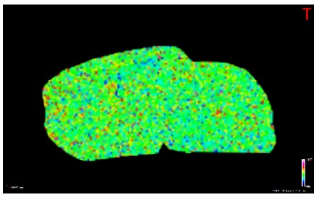 | 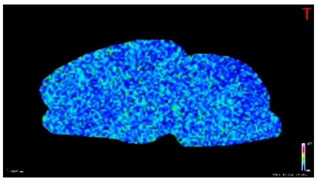 | 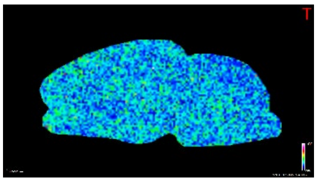 |
| **Ndufs6(21–116)** | **Basp1(79–193)** | **Ndufa2(2–99)@Ac** |
| 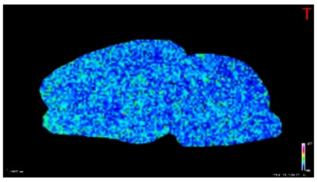 | 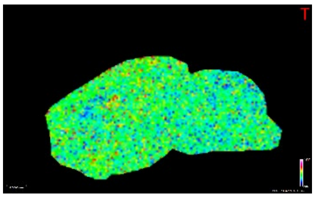 | 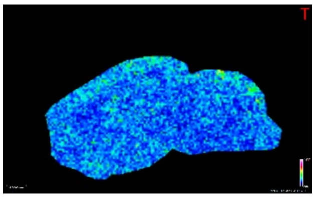 |
| **Hspe1(2–102)@Ac@[–35.7Da]** | **Hspe1(2–102)@Ac** | **Hspe1–rs1(2–102)@Ac** |
| 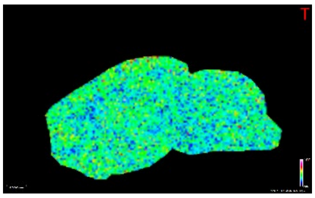 | 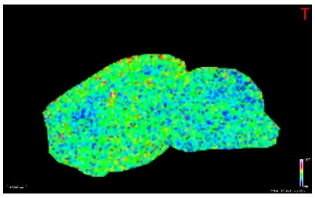 | 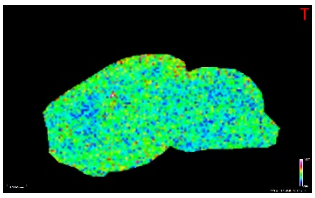 |
| **Dynlrb1(2–96)@Ac** | **Dynlrb1(2–96)@Ac@[+15.98Da]** | **Rpl37(2–97)** |

| | | |
|---|---|---|
| 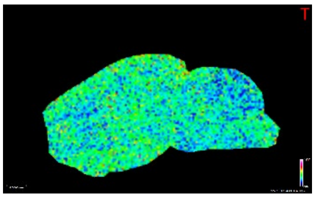 | 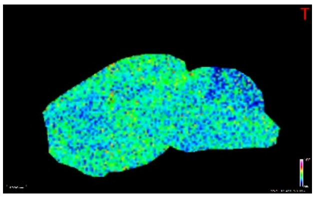 | 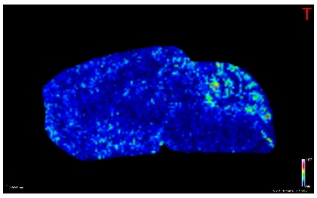 |
| Mrps36(2–102)@Ac | S100a13(2–98) | Ptms(2–97)@Ac |
| 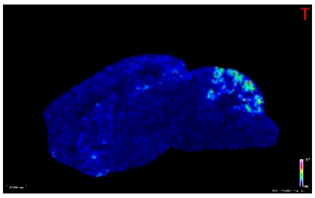 | 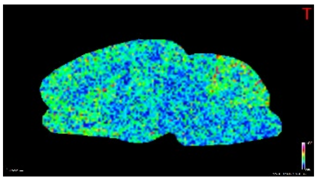 | 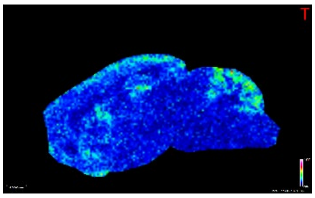 |
| S100a13(2–98)@Ac | Ptms(2–97)@Ac@Phospho | S100a13(1–98) |
| 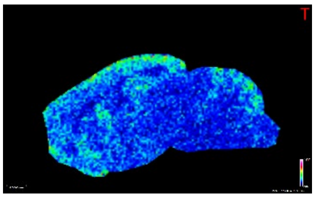 | 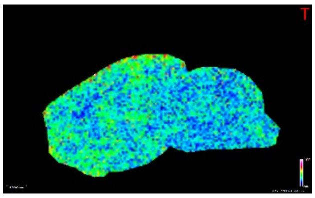 | 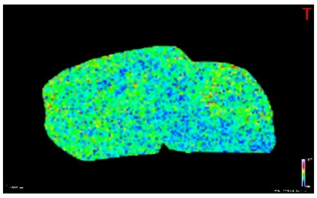 |
| Mrps36(2–102)@[+210.22Da] | Ptms(2–99)@Ac | Mbp(2–101)@Ac |
| 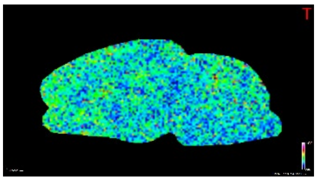 | 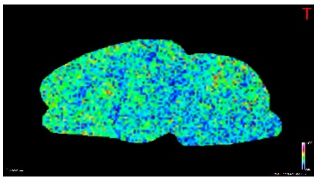 | 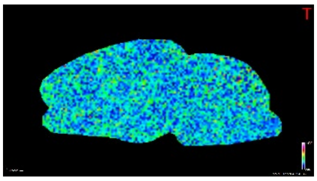 |
| H4c1(1–103)@[–60.97Da] | Ptms(2–101)@Ac | H4c1(2–103)@Ac@[+68.05Da] |
| 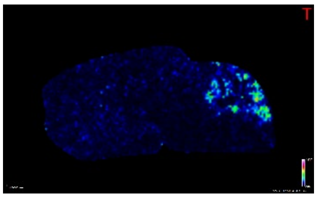 | 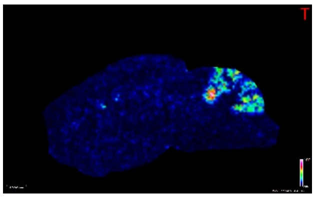 | 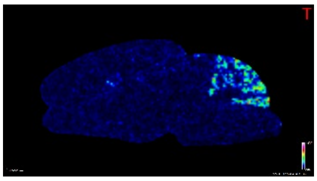 |
| H4c1(1–103)@Dimethyl | H4c1(1–103)@Ac@Methyl | Mbp(2–101)@Ac@[+220.52Da] |
| 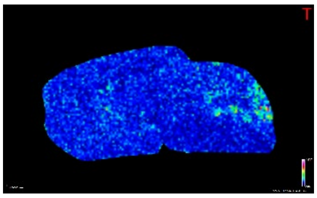 | 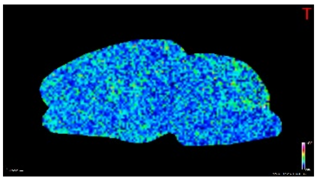 | 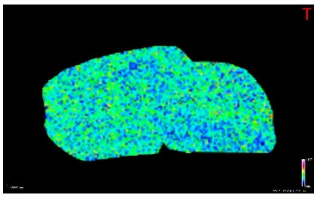 |

| | | |
|---|---|---|
| **H2aj(2–111)@Ac@[–470.45Da]** | **Basp1(1–109)@[+79.16Da]** | **Basp1(2–109)@Myristoyl** |
| 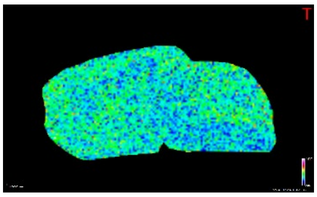 | 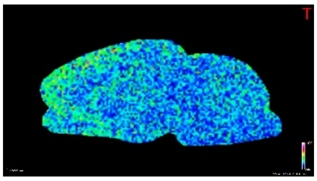 | 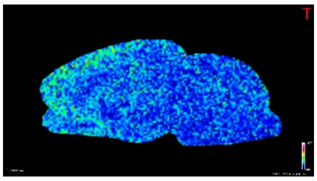 |
| **Gap43(112–227)@[–152.02Da]** | **Fkbp1a(2–108)** | **Fkbp1a(2–108)@Ac** |
| 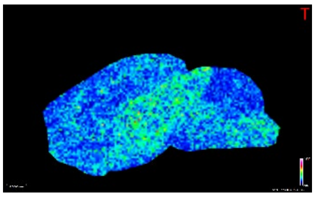 | 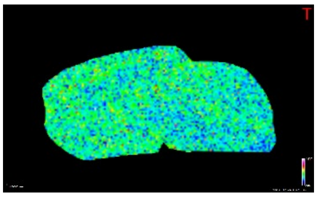 | 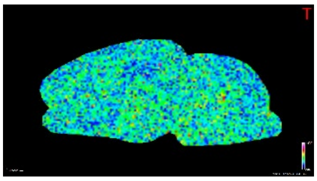 |
| **Pvalb(2–110)@Ac** | **Pvalb(2–110)@Ac@Oxi** | **Fkbp1a(2–108)@Ac*2** |
| 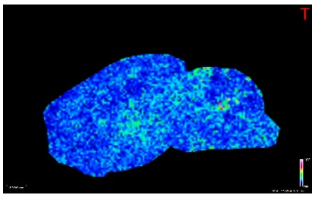 | 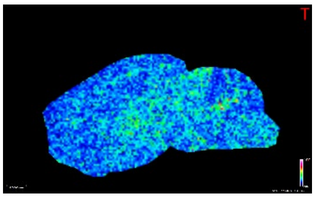 | 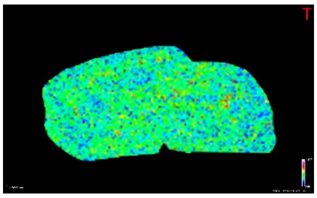 |
| **Fkbp1a(2–108)@Succinyl** | **Ptma(2–111)@Ac** | **Ptma(2–111)@Phospho** |
| 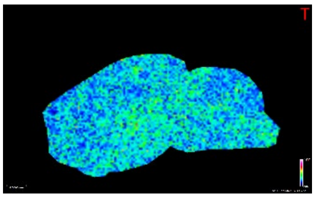 | 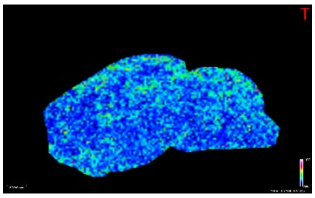 | 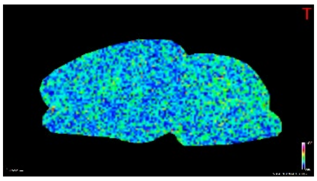 |
| **Gap43(1–111)@Ac@[+423.75Da]** | **Cox5a(38–146)** | **Cox5a(37–146)@[–148.07Da]** |
| 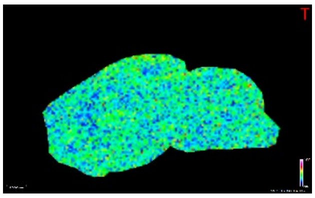 | 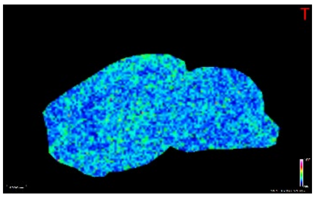 | 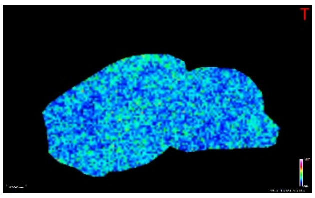 |
| **Cox5a(41–146)@Ac** | **Ndufa7(2–113)@Ac** | **Ndufa7(2–113)@Ac@[+14.15Da]** |

| | | |
|---|---|---|
| 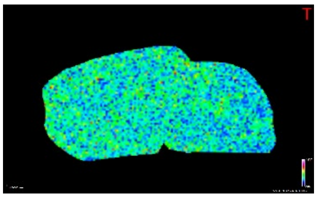 | 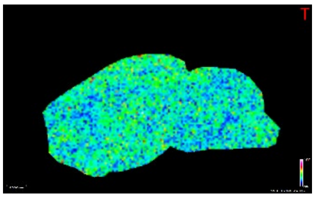 | 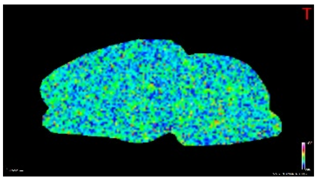 |
| Ndufa7(2–113)@Ac@[+17.32Da] | Ndufs5(2–106) | Cox5a(38–146)@[+114.84Da] |
| 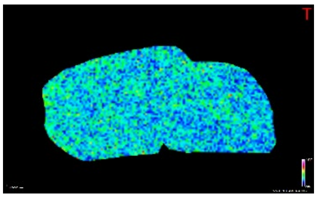 | 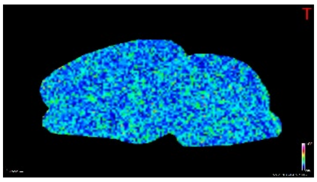 | 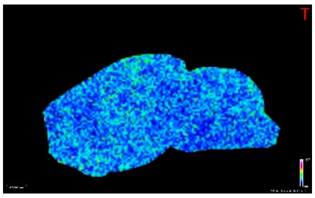 |
| Ndufs5(2–106)@Ac | Ndufa7(2–113)@Ac@Phospho | Vamp2–2(2–116)@Ac |
| 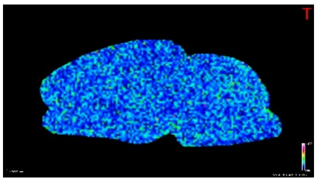 | 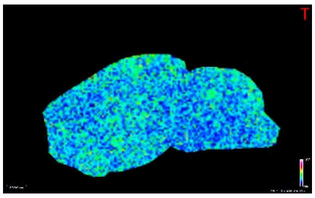 | 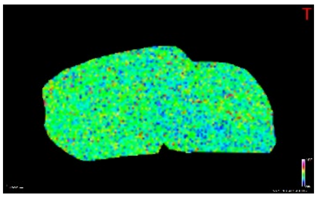 |
| Vamp2(2–116)@Ac | Pex3(1–107) | Basp1(90–226) |
| 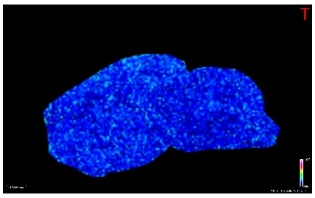 | 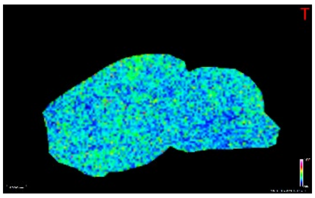 | 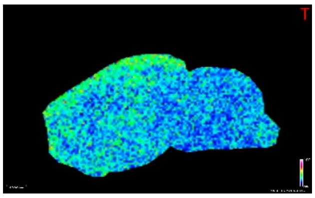 |
| Prrt2(2–125)@Ac@[–0.99Da] | Mbp(2–191)@Ac@Phospho | Calm2(1–113)@[+131.28Da] |
| 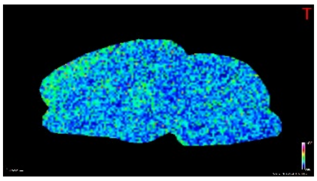 | 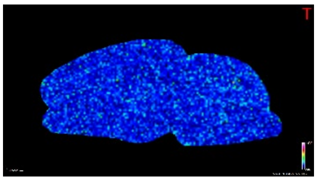 | 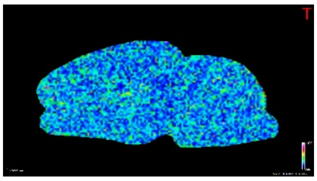 |
| Basp1(87–226) | Krtap12–1(1–130)@[–28.47Da] | H2az2(2–128) |
| 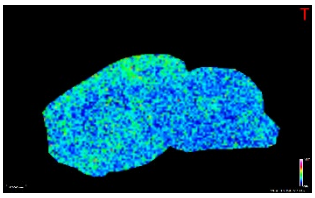 | 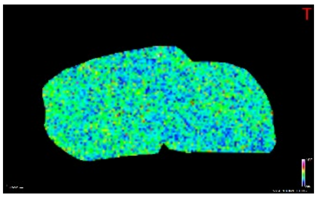 | 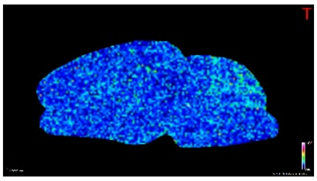 |

| H2az1(2–128) | Uqcrb(2–111)@Ac | Atp6v1g2(2–118) |
| --- | --- | --- |
| 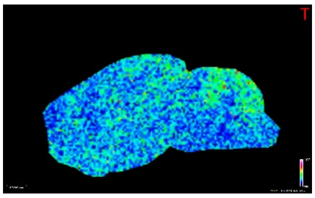 | 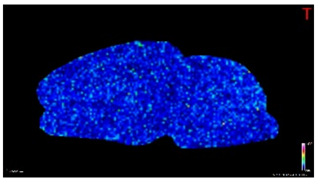 | 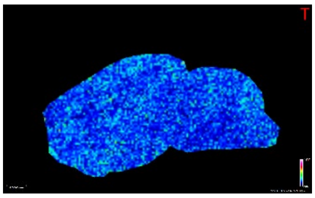 |
| Atp6v1g2(2–118)@Ac | Atp6v1g2(2–118)@Ac@Methyl | Atp6v1g1(2–118) |
| 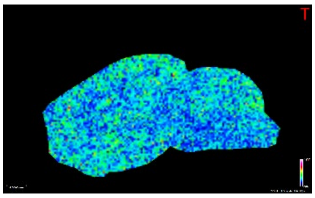 | 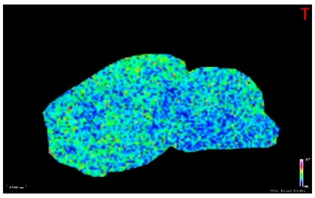 | 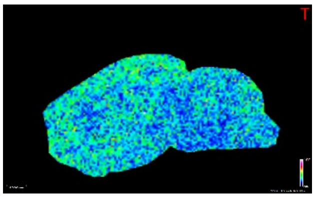 |
| Atp6v1g2(2–118)@Ac@Oxi | Atp6v1g1(2–118) | Atp6v1g1(2–118)@Ac |
| 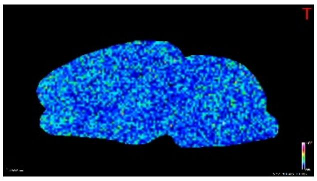 | 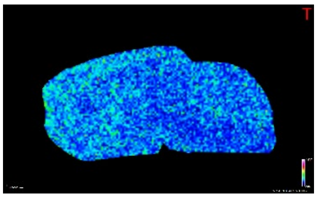 | 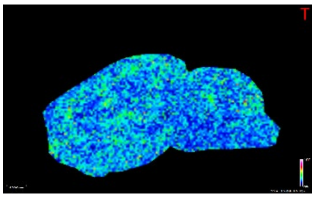 |
| Atp6v1g2(1–118) | Hint1(2–126)@Ac | Atp6v1g2(1–118)@Ac |
| 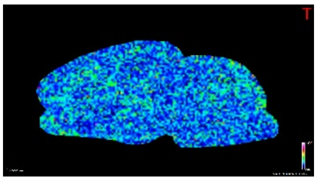 | 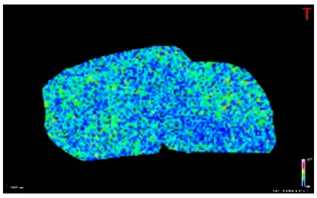 | 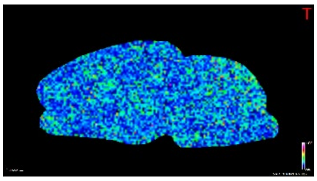 |
| Hist1h2bj(1–123) | Mbp(2–122)@Ac | Mbp(2–128)@Ac@[–370.2Da] |
| 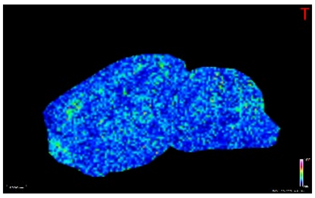 | 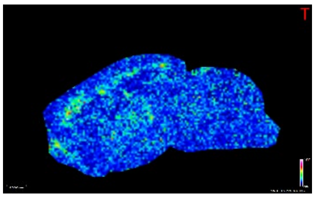 | 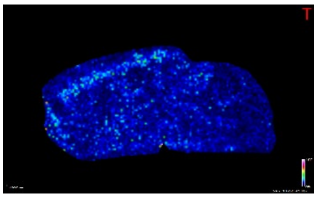 |
| H2bc4(2–126) | H2bc3(2–126)@[–42.67Da] | H2bc9(2–126)@[–10.88Da] |
| 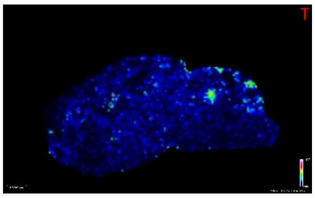 | 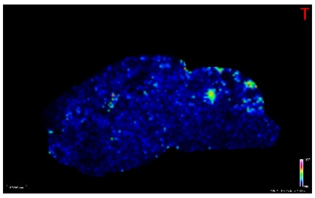 | 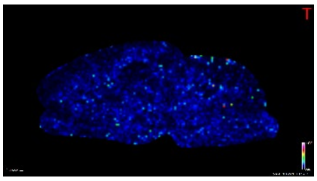 |

| | | |
|---|---|---|
| **H2bc7(2–126)@[–17.95Da]** | **H2bc4(2–126)@Oxi** | **H2bc4(2–126)@[+28.4Da]** |
| 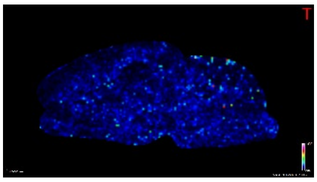 | 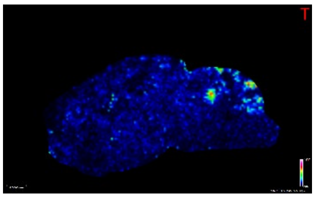 | 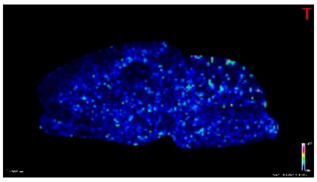 |
| **Mbp(2–126)@Ac** | **H2bc9(2–126)@[+29Da]** | **Mbp(2–126)@Ac@Methyl** |
| 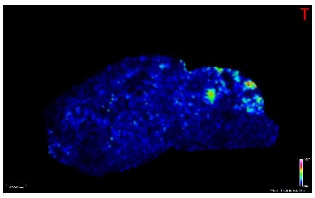 | 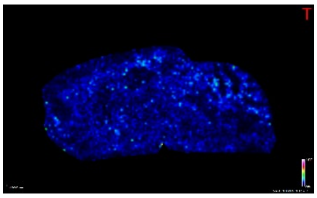 | 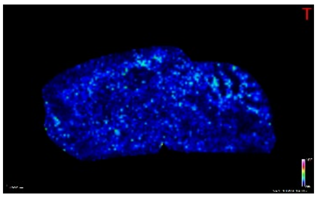 |
| **H2bc9(1–126)@Ac@[–133.1Da]** | **Mbp(2–128)@Ac@[–283.16Da]** | **H2bc14(2–126)@[+52.9Da]** |
| 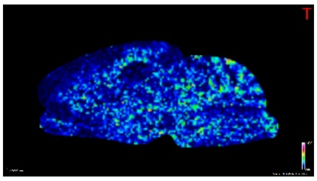 | 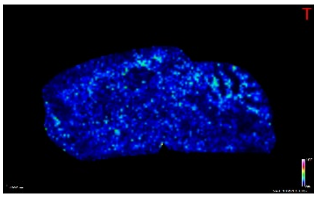 | 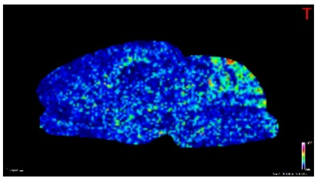 |
| **H2bc4(2–126)@Ac@Butanoyl** | **Hist2h2bb(2–126)@Ac@Crotonyl** | **Mbp(2–128)@Ac@[–218.23Da]** |
| 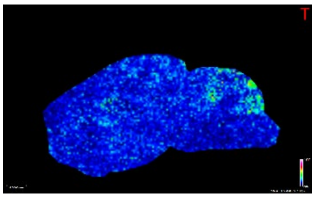 | 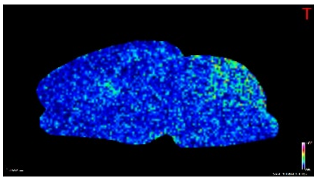 | 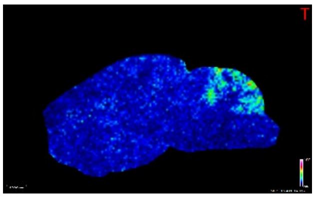 |
| **H2bc4(1–126)** | **Mbp(2–128)@Ac@[–203.11Da]** | **Mbp(2–127)@Ac** |
| 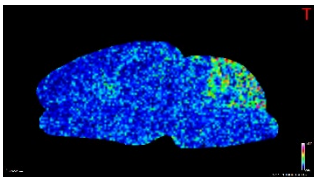 | 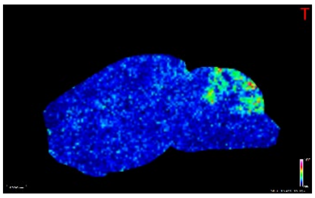 | 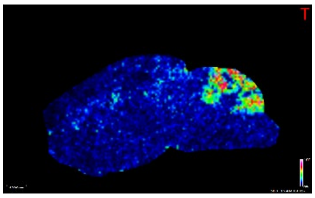 |
| **H2aj(2–129)@Ac@Methyl** | **Mbp(2–127)@Ac@Methyl** | **Hist2h2aa1(2–130)@Ac** |

| | | |
|---|---|---|
| 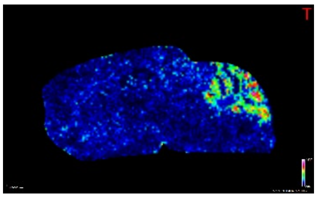 | 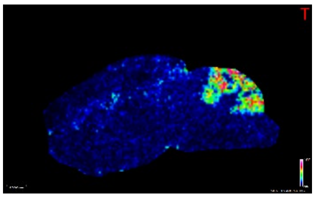 | 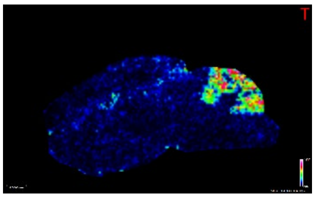 |
| **Hist2h2aa1(2–130)@Ac@[+4.25Da]** | **H2ac25(2–130)@Ac** | **H2ac4(2–130)@Ac** |
| 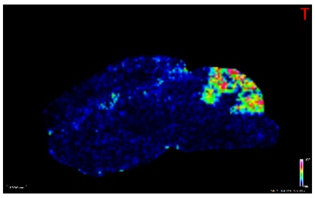 | 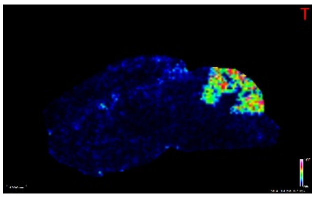 | 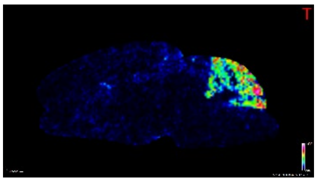 |
| **Dbi(9–135)@[–183.03Da]** | **Mbp(2–128)@Ac@[–62.07Da]** | **Sncb(1–133)@Ac** |
| 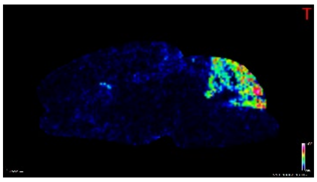 | 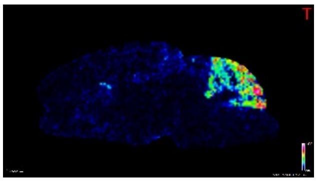 | 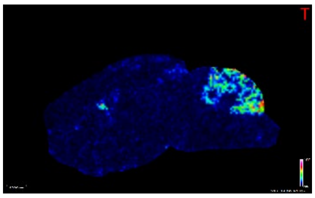 |
| **Sncb(1–133)@[+58.02Da]** | **Sncb(1–133)@Ac@[+16.31Da]** | **Mbp(2–128)@Ac** |
| 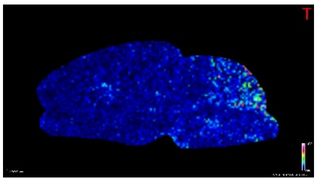 | 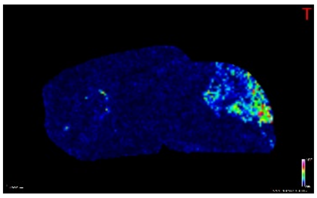 | 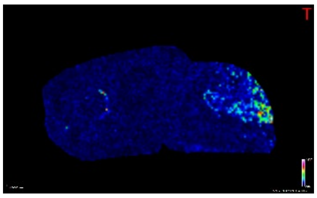 |
| **Sncb(1–133)** | **Mbp(2–128)@Ac@[+14.01Da]** | **Sncb(1–133)@Ac@[+52.27Da]** |
| 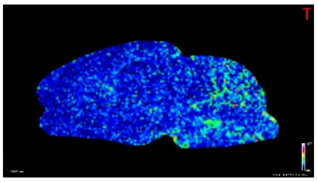 | 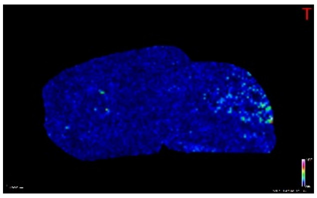 | 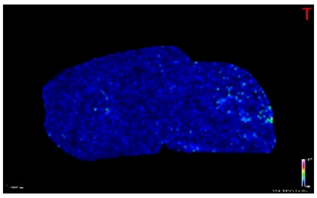 |
| **Sncb(1–133)@Ac@[+57.26Da]** | **Rpl31(4–125)** | **Rida(2–135)@Ac** |

| | | |
|---|---|---|
| 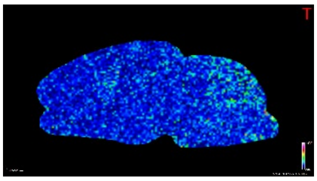 | 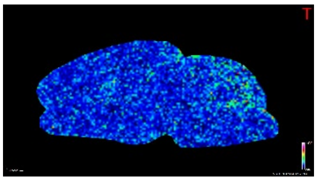 | 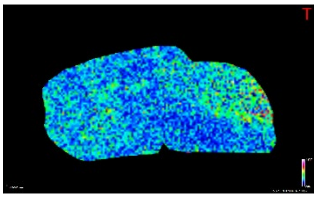 |
| Sncb(1–133)@Ac@Phospho | Mbp(2–128)@Ac@Phospho | Mbp(2–128)@Ac@[+94.03Da] |
| 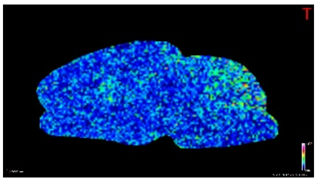 | 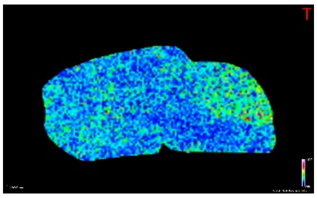 | 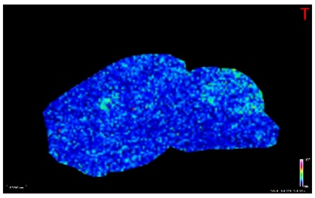 |
| Mbp(2–128)@Ac@[+109.08Da] | Mbp(2–128)@Ac@[+127.09Da] | Mbp(2–128)@Ac@[+173.98Da] |
| 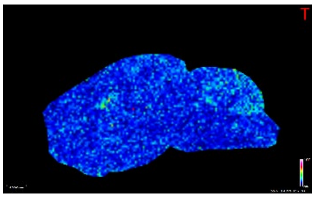 | 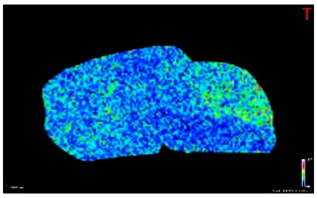 | 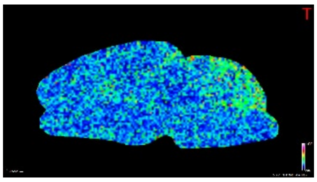 |
| Mbp(2–128)@Ac@[+207.32Da] | Rpl35(2–123) | Hist1h2bp(2–126)@Butanoyl@ADP–ribosyl |
| 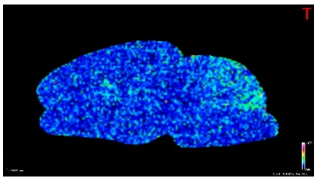 | 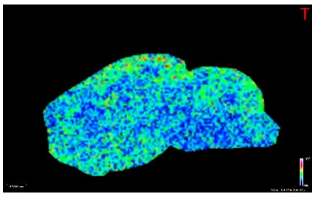 | 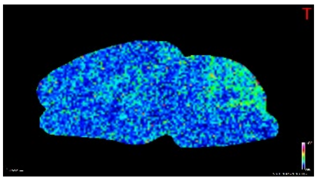 |
| H2bc4(1–126)@Ac@ADP–ribosyl | H2bc7(1–126)@Ac@ADP–ribosyl | Snca(1–140)@Ac |
| 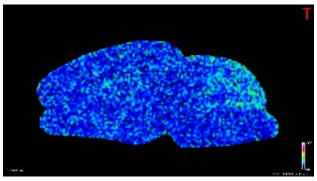 | 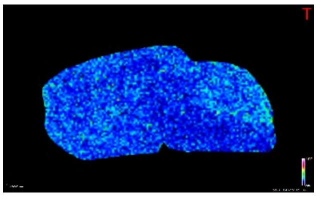 | 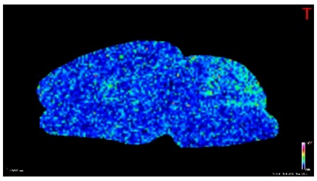 |
| Snca(1–140)@Ac@[+16.55Da] | Snca(1–140)@Ac@[+30.34Da] | Snca(1–140)@Ac@[+33.55Da] |

| | | |
|---|---|---|
| 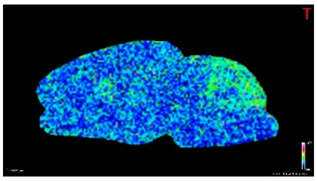 | 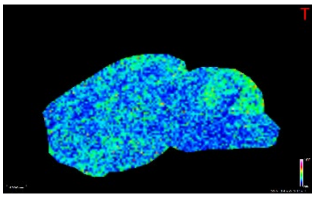 | 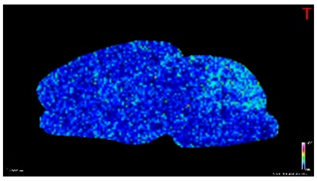 |
| Sncb(1–133)@Ac@[+480.64Da] | Snca(1–140)@Ac@[+47.52Da] | H2bc14(1–126)@Ac@ADP–ribosyl@Butanoyl |
| 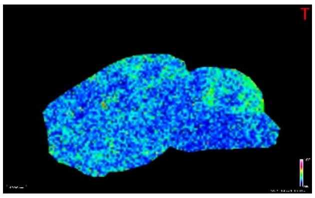 | 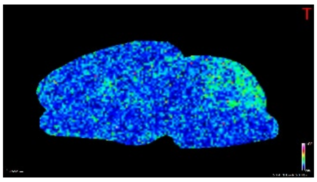 | 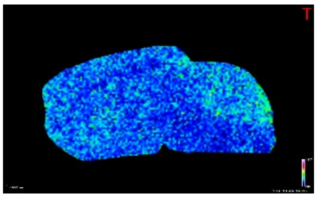 |
| Mbp(2–128)@Ac@[+470.22Da] | Hist1h2bp(1–126)@Ac*2@ADP–ribosyl | Mbp(2–140)@Ac@[–471.25Da] |
| 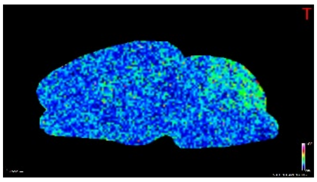 | 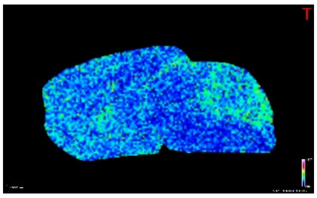 | 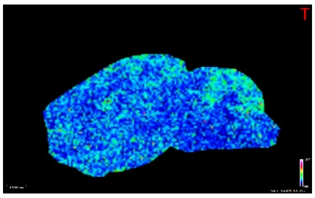 |
| Mbp(2–138)@Ac@[–42.88Da] | H2ax(2–143)@Ac | Hbb–bs(3–147)@[–456.35Da] |
| 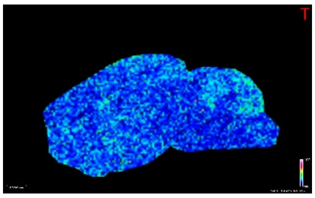 | 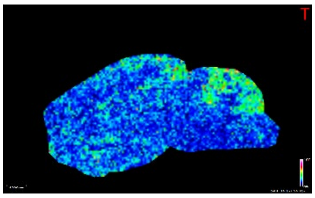 | 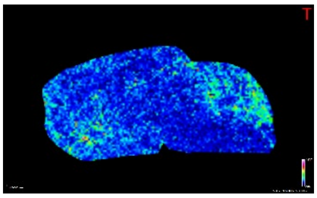 |
| H2ax(2–143)@Ac*2 | Cplx1(1–134) | Dbi(2–135)@Ac |
| 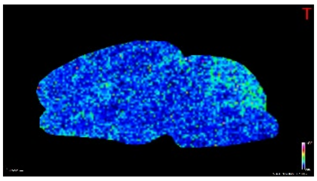 | 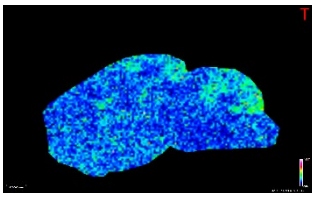 | 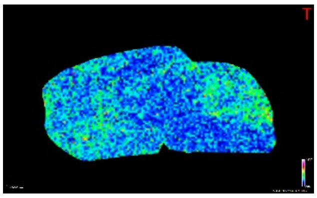 |
| Cplx1(1–134)@Ac | Cplx1(1–134)@Ac@[+18.01Da] | Cplx1(1–134)@Ac@[+31.98Da] |
| 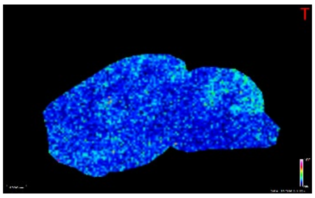 | 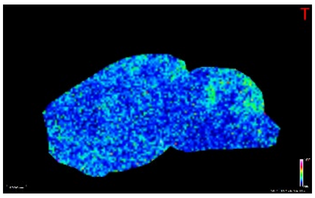 | 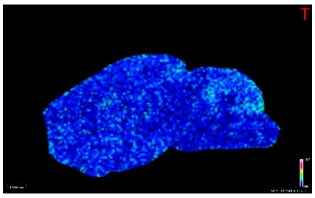 |

| | | |
|---|---|---|
| H3f4(2–136) | H3–3a(1–136)@Ac@[–44.83Da] | Cplx2(2–134)@Phospho |
| 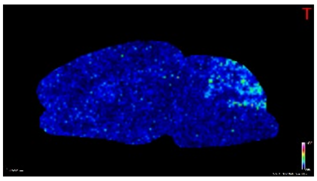 | 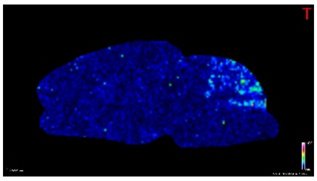 | 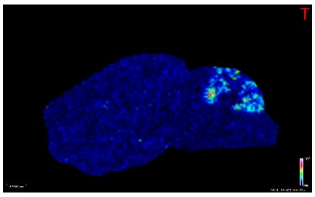 |
| H3–3a(1–136)@Ac | Cplx2(1–134)@Ac@[+32Da] | Spmip11(1–131) |
| 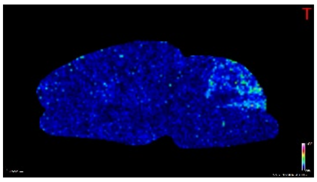 | 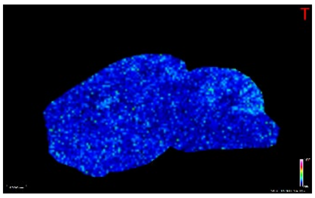 | 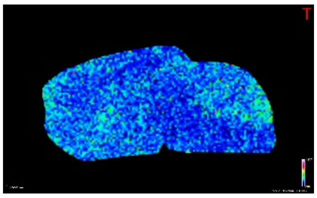 |
| Cplx2(1–134)@Ac@Phospho | Sod1(2–154)@Ac | Sod1(2–154)@Ac*2 |
| 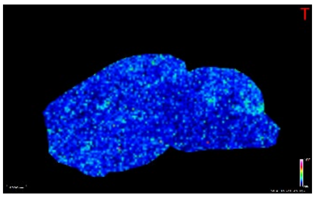 | 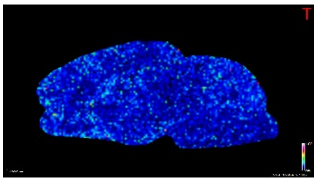 | 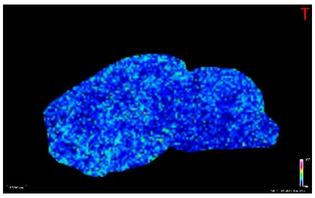 |
| Aβ(1–42) | Tmsb4x(13–44) | Snca(95–140)@[16.18Da] |
| 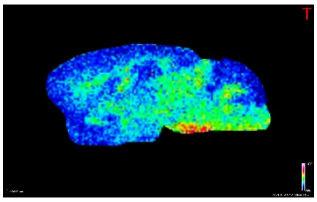 | 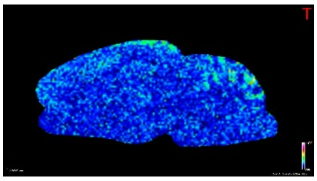 | 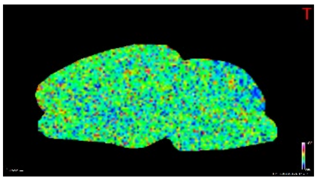 |
| Snca(1–67)@Ac@Oxi | Tmsb10(2–42)@Ac | Tmsb4x(2–39)@Ac |
| 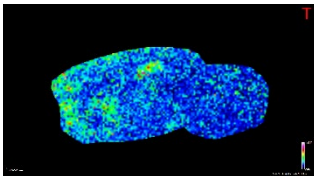 | 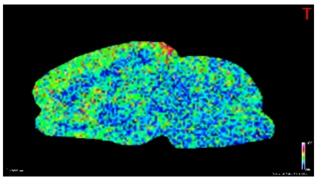 | 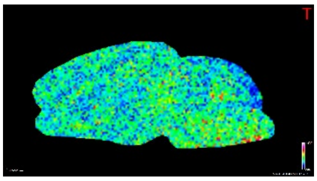 |
